# A Review of SHV Extended-Spectrum β-Lactamases: Neglected Yet Ubiquitous

**DOI:** 10.3389/fmicb.2016.01374

**Published:** 2016-09-05

**Authors:** Apostolos Liakopoulos, Dik Mevius, Daniela Ceccarelli

**Affiliations:** ^1^Department of Bacteriology and Epidemiology, Central Veterinary Institute of Wageningen URLelystad, Netherlands; ^2^Faculty of Veterinary Medicine, Utrecht UniversityUtrecht, Netherlands

**Keywords:** β-lactamase, ESBL, *bla*_SHV_, SHV-2, SHV-5, SHV-12, plasmid, Enterobacteriaceae

## Abstract

β-lactamases are the primary cause of resistance to β-lactams among members of the family Enterobacteriaceae. SHV enzymes have emerged in Enterobacteriaceae causing infections in health care in the last decades of the Twentieth century, and they are now observed in isolates in different epidemiological settings both in human, animal and the environment. Likely originated from a chromosomal penicillinase of *Klebsiella pneumoniae*, SHV β-lactamases currently encompass a large number of allelic variants including extended-spectrum β-lactamases (ESBL), non-ESBL and several not classified variants. SHV enzymes have evolved from a narrow- to an extended-spectrum of hydrolyzing activity, including monobactams and carbapenems, as a result of amino acid changes that altered the configuration around the active site of the β -lactamases. SHV-ESBLs are usually encoded by self-transmissible plasmids that frequently carry resistance genes to other drug classes and have become widespread throughout the world in several Enterobacteriaceae, emphasizing their clinical significance.

## Introduction

Thanks to their ability to inhibit cell wall biosynthesis, β-lactams remained the first-line defense against bacterial infections for over 20 years, before resistant bacteria appeared in clinical practice.

Resistance to this class of drugs can be the result of antibiotic target site alteration, prevention of antibiotic access by altered permeability or forced efflux, or antibiotic degradation (Wilke et al., 2005). The latter, represents the primary resistance mechanism in Gram-negative bacteria producing β-lactamase enzymes able to covalently bind the carbonyl moiety of the β-lactam ring and hydrolyze its amide bond (Fisher et al., 2005). Naturally occurring chromosomally located β-lactamases are quite common in Gram-negative bacteria; likely evolved from penicillin-binding proteins, when produced in small quantity they do not significantly contribute to antibiotic resistance. It was the appearance of the first plasmid-mediated β-lactamase TEM-1 (Datta and Kontomichalou, 1965) to designate the beginning of an unstoppable phenomenon in the 1960s. Ever since, the introduction of new natural or synthetic drugs to replace old ones in an attempt to limit the insurgence of antibiotic resistant bacteria triggered a chain reaction providing bacteria with a constant selective pressure driving the expansion of different resistance mechanisms (Medeiros, 1997).

In recent years β-lactamases have extensively diversified in response to the clinical use of new generations of β-lactams (penicillin, cephalosporins, carbapenems, and monobactams) leading to the need of classification schemes. Based on primary structure (Ambler, 1980), enzymatic properties and biochemical attributes (Bush et al., 1995), and the increasingly available amino acid sequences (Bush and Jacoby, 2010) four major classes (A, B, C, D) can be acknowledged. Serine β-lactamases belonging to class A are the most abundant (Philippon et al., 2016), with more than 500 enzymes, including the most clinically significant extended spectrum β-lactamases (ESBL) variants, i.e., CTX-M-, TEM-, and SHV-type enzymes (Bush and Fisher, 2011).

Although, SHV enzymes did not undergo the explosive dissemination observed for CTX-M-type variants (Canton et al., 2012), in recent years they have been found in several Enterobacteriaceae outside of the typical clinical hosts *Klebsiella pneumoniae* and *Escherichia coli*, with a rising allele variability (http://www.lahey.org/studies), and in different environmental niches. Many admirable works describing the biochemistry, the genetics and the evolution of SHV β-lactamases have appeared over the last years. The aim of this review is to provide the readers with an updated overview on SHV β-lactamases, their amino acid variants and spectrum of activity, and to describe the occurrence of plasmid-associated SHV enzymes in Enterobacteriaceae and their epidemiological significance.

## Origin and diversity of the SHV family

The first *bla*_SHV-1_ gene was identified in the 1970s in *E. coli* (Pitton, 1972). The encoded enzyme SHV−1 (sulfhydryl reagent variable) proved its activity against penicillins and first generation cephalosporins (Matthew et al., 1979) and was confirmed part of the conjugative plasmid p453 (Barthélémy et al., 1988; Table 1). The most likely ancestor of the plasmid-mediated SHV−1 is a chromosomal species-specific penicillinase detected in fecal *K. pneumoniae* isolates from neonates (Haeggman et al., 1997). The enzyme showed a typical antibiogram with penicillin rather than cephalosporin resistance and a marked inhibition by clavulanic acid. How *bla*_SHV-1_ moved from the chromosome to the plasmid does not have a conclusive explanation since the proposed association with a transposable element (Nugent and Hedges, 1979) has not been confirmed.

**Table 1:** **SHV-type extended-spectrum β-lactamases**.

**Gene^§^**	**Accession Number**	**pI**	**Isolation**		**Bacterial Species**	**Genetic background**				**References**
			**Location**	Year^*^		**Genetic Location^¥^**	**Conjugative plasmid**	**Plasmid (Kb)**	**Other Ab genes**	
*bla*_SHV-1_^**^	AF148850	7.6	NA	1972	*E. coli*	p^453^	Yes	ND	ND	Pitton, 1972; Matthew et al., 1979
*bla*_SHV-2_	AF148851	7.6	Germany	1983	*K. ozaenae*	p^BP60^	Yes	45	ND	Kliebe et al., 1985
*bla*_SHV-2a_	X98102	7.6	Germany	1987–1988	*K. pneumoniae*	p^ZMP1^	Yes	66	ND	Podbielski et al., 1991
*bla*_SHV-3_	KX092356	7.0	France	1986	*K. pneumoniae*	p^UD18^	Yes	180	ND	Nicolas et al., 1989
*bla*_SHV-4_	NA	7.8	France	1987	*K. pneumoniae*	P	Yes	180	ND	Péduzzi et al., 1989; Arlet et al., 1990
*bla*_SHV-5_	X55640	8.2	Chile	1987	*K. pneumoniae*	p^AFF1^	No	150	ND	Gutmann et al., 1989
*bla*_SHV-6_	Y11069.1	7.6	France	1991	*K. pneumoniae*	p^SLH06^	Yes	180	ND	Arlet et al., 1991
*bla*_SHV-7_	U20270	7.6	USA	1993	*E. coli*	P	Yes	10	ND	Bradford et al., 1995
*bla*_SHV-8_	U92041	7.6	USA	1990	*E. coli*	C	–	–	–	Rasheed et al., 1997
*bla*_SHV-9_	S82452.1	8.2	Greece	1995	*E. coli; K. pneumoniae; S. marcescens*	p^K318-1^; p^E77-1^; p^S24-1^	Yes	ND	ND	Prinarakis et al., 1996
*bla*_SHV-11_^**^	X98101	8.2	Switzerland	1993–1995	*K. pneumoniae*	P	Yes	80	ND	Nüesch-Inderbinen et al., 1997
*bla*_SHV-12_	JX268741	8.2	Switzerland	1993–1995	*E. coli; K. pneumoniae*	P	Yes	80	ND	Nüesch-Inderbinen et al., 1997
*bla*_SHV-13_	AF164577	7.6	Netherlands	1994	*K. pneumoniae*	P	Yes	170	ND	Yuan et al., 2000
*bla*_SHV-15_	AJ011428.2	ND	India	1998	*E. coli*	ND	ND	ND	ND	http://www.lahey.org/studies/
*bla*_SHV-16_	AF072684.2	7.6	France	1996	*K. pneumoniae*	P	Yes	>100	–	Arpin et al., 2001
*bla*_SHV-18_	AF132290	7.8	USA	1994	*K. pneumoniae*	P	Yes	80	ND	Rasheed et al., 2000
*bla*_SHV-23_	AF117747	ND	South Africa	1990	*K. pneumoniae*	ND	ND	ND	ND	Essack et al., 2004
*bla*_SHV-24_	AB023477	7.5	Japan	1996	*E. coli*	p^CAZR001^	Yes	150	ND	Kurokawa et al., 2000
*bla*_SHV-27_	AF293345.1	8.2	Brazil	1999	*K. pneumoniae*	C	–	–	ND	Corkill et al., 2001
*bla*_SHV-30_	AY661885	6.7	USA	2003	*E. cloacae*	P	ND	9.4	AmpC, *bla*_TEM-1_ and *bla*_SHV-7_	Szabó et al., 2005
*bla*_SHV-31_	AY277255	7.8	Netherlands	2001	*K. pneumoniae*	C	–	–	–	Mazzariol et al., 2007
*bla*_SHV-34_	AY036620	ND	USA	1998–2000	*C. koseri; E. coli; K. pneumoniae*	p^OZ185^	Yes	>100	ND	Heritage et al., 2003
*bla*_SHV-38_	AY079099	7.6	France	2001	*K. pneumoniae*	C	–	–	–	Poirel et al., 2003
*bla*_SHV-40_	AF535128	7.6	Canada	1999–2000	*K. pneumoniae*	ND	ND	ND	ND	Mulvey et al., 2004
*bla*_SHV-41_	AF535129	7.6	Canada	1999–2000	*K. pneumoniae*	ND	ND	ND	ND	Mulvey et al., 2004
*bla*_SHV-42_	AF535130	7.6	Canada	1999–2000	*K. pneumoniae*	ND	ND	ND	ND	Mulvey et al., 2004
*bla*_SHV-45_	AF547625	8.2	Brazil	NA	*K. pneumoniae*	IncA/C	ND	97-145	*bla*_CTX-M-2_ and *bla*_SHV-27_	Dropa et al., 2015
*bla*_SHV-46_	AY210887	8.2	New York	1998	*K. oxytoca*	P	Yes	70	*bla*_TEM-1_; *bla*_OXY-2_; *bla*_KPC-2_; *bla*_OXA (?)_	Yigit et al., 2003
*bla*_SHV-55_	DQ054528	ND	Portugal	NA	*K. pneumoniae*	ND	No	–	TEM1	Mendonça et al., 2006
*bla*_SHV-57_	AY223863	8.3	Taiwan	1998	*E. coli*	p^MTY512^	Yes	40–60	ND	Ma et al., 2005
*bla*_SHV-64_	DQ174304	ND	China	2000–2002	*K. pneumoniae*	ND	ND	ND	ND	Zuo et al., 2006
*bla*_SHV-66_	DQ174306	ND	China	2000–2002	*K. pneumoniae*	ND	ND	ND	ND	Zuo et al., 2006
*bla*_SHV-70_	DQ013287	7.6	China	2003–2004	*E. cloacae*	p^EC04^	Yes	ND	ND	Ling et al., 2006
*bla*_SHV-86_	DQ328802	8.2	Colombia	2003	*K. pneumoniae*	P	Yes	ND	ND	Espinal et al., 2010
*bla*_SHV-90_	NA	8.2	Portugal	2003	*K. pneumoniae*	ND	ND	ND	ND	Machado et al., 2007
*bla*_SHV-91_	NA	7.6	Portugal	2003	*K. pneumoniae*	ND	ND	ND	ND	Machado et al., 2007
*bla*_SHV-98_	AM941844	7.6	Algeria	2005	*K. pneumoniae*	ND	ND	ND	ND	Ramdani-Bouguessa et al., 2011
*bla*_SHV-99_	AM941845	7.8	Algeria	2005	*K. pneumoniae*	ND	ND	ND	ND	Ramdani-Bouguessa et al., 2011
*bla*_SHV-100_	AM941846	7.2	Algeria	2005	*K. pneumoniae*	ND	ND	ND	ND	Ramdani-Bouguessa et al., 2011
*bla*_SHV-102_	EU024485	ND	Spain	2003–2004	*E. coli*	ND	ND	ND	ND	Vinué et al., 2008
*bla*_SHV-104_	EU274581	7,3/8,6	Tunisia	2004	*K. pneumoniae*	p^ML2011^	Yes	50	ND	Ben Achour et al., 2014
*bla*_SHV-105_	FJ194944	ND	USA	NA	*K. pneumoniae*	ND	ND	ND	*bla*_SHV-1_; *bla*_SHV-5_	Jones et al., 2009
*bla*_SHV-106_	AM941847	7.6	Portugal	1999	*K. pneumoniae*	ND	ND	ND	*bla*_TEM-1_; *bla*_CTX-M-32_	Mendonça et al., 2009
*bla*_SHV-128_	GU932590	8.6	Tunisia	2009	*E. cloacae*	IncFII (IS*26*)	Yes	100	ND	Bourouis et al., 2015
*bla*_SHV-129_	GU827715	ND	Italy	2008	*E. coli*	p^Ec6-66^	ND	ND	ND	Lascols et al., 2012
*bla*_SHV-134_	HM559945	ND	Spain	2009	*K. pneumoniae*	IncFIIA (IS*26*)	Yes	75	*bla*_VIM-1_; *aac*(6′)-*Ib; dhfrII; aadA1; catB2; bla*_TEM-1_; *aac(3′)-Iia*	Sánchez-Romero et al., 2012
*bla*_SHV-183_	HG934764	ND	NA	NA	*E. cloacae*	ND	ND	ND	ND	http://www.lahey.org/studies/

§ *Gene bla_SHV−115_ was not included in the table because no information is available (http://www.lahey.org/studies)*.

* *Isolation or first description*.

** *Non ESBL genes bla_SHV-1_ and bla_SHV-11_ are provided as reference*.

¥ *P, plasmid; C, Chromosome; when known plasmid name or Inc/rep group, and Insertion Sequences are indicated*.

*NA, not available; ND, not determined*.

As of today, 189^1^ SHV allelic variants have been described, having developed resistance to 3rd generation cephalosporin (Tzouvelekis and Bonomo, 1999), monobactam and carbapenems (Poirel et al., 2003). Only a small proportion is biochemically and/or genetically characterized (http://www.lahey.org/studies). SHV β-lactamases can be divided into three subgroups on the basis of molecular characteristics or functional properties: (i) subgroup 2b (*n* = 37), able to hydrolyze penicillins and early cephalosporins (cephaloridine and cephalothin) and strongly inhibited by clavulanic acid and tazobactam; (ii) subgroup 2br (*n* = 7), broad-spectrum β-lactamases that acquired resistance to clavulanic acid; and (iii) subgroup 2be (*n* = 46), comprises ESBLs that can also hydrolyze one or more oxyimino β-lactams (cefotaxime, ceftazidime, and aztreonam). More than half of these variants (*n* = 99) has not been classified yet due to absence of biochemical characterization.

**Figure 1:**
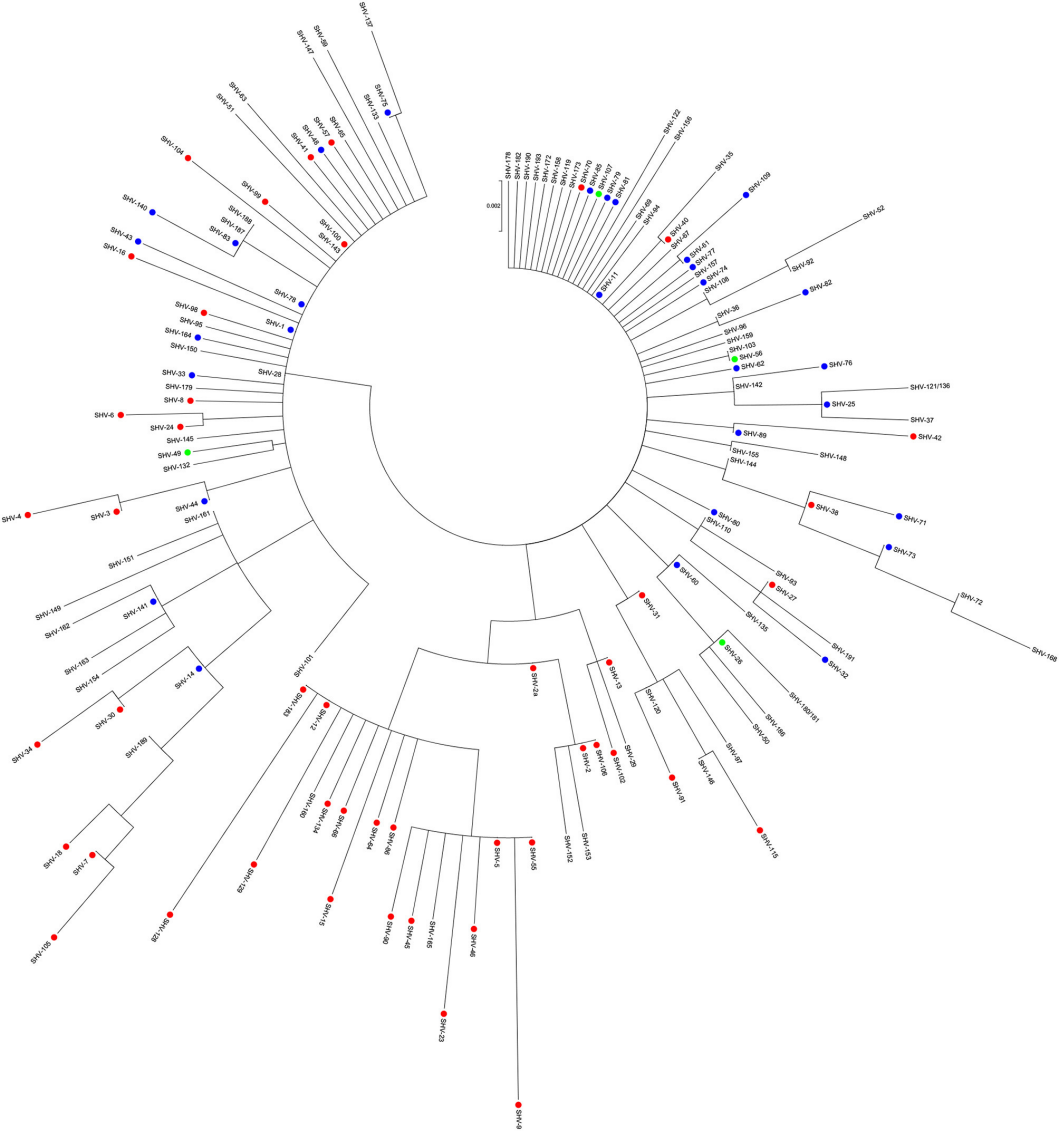
**Maximum likelihood amino acid tree of 149 SHV-type β-lactamases**. Variants whose sequence has not been released in GenBank as of July 2016, that show partial sequence or are identical to others (http://www.lahey.org/studies/) were not included in the analysis. SHV-180 and SHV-181 share the same sequence as well as SHV-121 and SHV-136. The tree was implemented in Mega version 6.06 (Tamura et al., 2013). Solid circles represent: red, extended-spectrum β-lactamases (2be; *n* = 46); green, broad-spectrum β-lactamases (2br, *n* = 5); and blue, penicillinases (2b, *n* = 30). Unclassified alleles are reported in black (*n* = 68).

Figure 1 illustrates a phylogenetic analysis of 149 out of the 189 SHV β-lactamase variants whose amino acid sequences were available online (http://www.lahey.org/studies), as of July 2016. Unlike other β-lactamase families (D'andrea et al., 2013; Evans and Amyes, 2014), there is no clear clustering of the different subgroups, as also mirrored by gene based analysis (Supplementary Figure S1). Among the majority of unclassified variants, subgroup 2b and the few 2br variants are scattered all over the tree. Subgroup 2be showed clustering of most of the ESBL variants (including SHV-2a, SHV-5, and SHV-12), together with few non-classified enzymes (SHV−29, SHV−152, SHV−153, SHV−160, and SHV−165). It has been proposed that SHV β-lactamases descended from an unidentified ancestor holding an extended spectrum phenotype (2be) and that subgroup 2b derived from it (Hall and Barlow, 2004). Our analysis showed that several of SHV ESBL variants were scattered along the tree with short branch lengths with neighboring 2b or unknown variants within the SHV phylogeny (i.e., SHV−40, SHV−11, and SHV−35; Figure 1), supporting the hypothesis that they evolved from multiple variants, probably within the antibiotic era. Among the non-ESBL variants, *bla*_SHV-11_ represents one of the most successful and, together with *bla*_SHV-1_, the likely source of evolution for the existing SHV ESBL variants. *bla*_SHV-11_ was first identified as plasmid-encoded in clinical *K. pneumoniae* from Switzerland (Nüesch-Inderbinen et al., 1997) and ever since has been isolated worldwide.

Although, nearly displaced, together with TEM, by CTX-M enzymes over the years (Canton et al., 2012), 46 ESBL *bla*_SHV_ genes have been described so far (Table 1). The first report of SHV-mediated resistance to third-generation cephalosporins was in 1983 with the isolation and characterization of *bla*_SHV-2_, encoded by plasmid pBP60 in a German clinical isolate of *Klebsiella ozaenae* and showing only a few nucleotide mismatches with *bla*_SHV-1_ (Kliebe et al., 1985). In a few years four other ESBL variants were identified as plasmid-encoded in clinical *K. pneumoniae*, showing variable gene homologies with the *bla*_SHV-1_ and *bla*_SHV-2_ sequences (50–90%): *bla*_SHV-2*a*_ encoded by conjugative plasmid pZMP1 (Podbielski et al., 1991); *bla*_SHV-3_ on pUD18 (Nicolas et al., 1989); *bla*_SHV-4_, widely disseminated from France as a result of a single *K. pneumoniae* clone diffusion (Arlet et al., 1990, 1994); and *bla*_SHV-5_ able to hydrolyze broad-spectrum cephalosporins and monobactams (Gutmann et al., 1989). Of these first variants, the most epidemiologically successful were *bla*_SHV-2*a*_ and *bla*_SHV-5_, which will be further discussed, together with *bla*_SHV-2_ and *bla*_SHV-12_, in a dedicated paragraph (Section Expansion toward New Ecological Niches). Interestingly, *bla*_SHV-3_ and *bla*_SHV-4_ have been only sporadically detected since their first description. *bla*_SHV-3_ seems to be geographically restricted to the USA where it was detected in *E. coli* of animal origin, associated with other antibiotic resistance genes such as *bla*_CTX-*M*-15_, *bla*_CTX-*M*-24_, *bla*_CMY-2_, and/or *bla*_TEM-1_ (Shaheen et al., 2011). *bla*_SHV-4_ was identified also in *Enterobacter aerogenes* and *Citrobacter diversus* in different countries (Arpin et al., 1996; El Harrif-Heraud et al., 1997; Pitout et al., 1998).

The last two decades witnessed the appearance of several new variants (*bla*_SHV-7_, *bla*_SHV-8_, *bla*_SHV-9_, *bla*_SHV-31_, *bla*_SHV-38_, *bla*_SHV-40_, *bla*_SHV-41_, and *bla*_SHV-42_) whose dissemination was restricted to limited cases (Supplementary Table S1). A few variants seem to be geographically constrained: (i) *bla*_SHV-106_, only described in Portuguese isolates of *K. pneumoniae* together with *bla*_TEM-1_, and/or *bla*_CTX-*M*-32_ (Mendonça et al., 2009); (ii) *bla*_SHV-55_, in Portugal (Mendonça et al., 2006; Machado et al., 2007) and recently in Brazil (Dropa et al., 2015); and (iii) *bla*_SHV-57_, in *E. coli* isolates from Taiwan and China (Ma et al., 2005; Tian et al., 2012). A variant worth to mention is *bla*_SHV-27_ (Corkill et al., 2001), that has been detected on different plasmids in *E. coli, K. pneumoniae* and *Enterobacter cloacae*, associated with a vast array of antibiotic resistance genes (*bla*_DHA−1_, *bla*_TEM-1_, *bla*_TEM−1b_, *bla*_CMY-2_, *bla*_IMP_, *bla*_CTX-*M*-14_, *bla*_CTX-*M*-15_, *bla*_SHV-12_, *bla*_SHV-45_, *bla*_OXA-1_, *dfrA5, ereA2*; Muratani et al., 2006; Abbassi et al., 2008; Kiratisin et al., 2008; Duval et al., 2009; Hammami et al., 2011).

Most of SHV ESBLs (25 out of 46) are unique cases, with only one report so far. Seventeen variants are exclusively found in clinical *K. pneumoniae*: *bla*_SHV-6_, *bla*_SHV-13_, *bla*_SHV-16_, *bla*_SHV-18_, *bla*_SHV-23_, *bla*_SHV-45_, *bla*_SHV-64_,*bla*_SHV-66_, *bla*_SHV-86_, *bla*_SHV-90_, *bla*_SHV-91_, *bla*_SHV-98_, *bla*_SHV-99_, *bla*_SHV-100_,*bla*_SHV−104_,*bla*_SHV-105_, and *bla*_SHV-134_. These variants have been described worldwide (Brazil, Portugal, Algeria, USA, Tunisia, Netherlands, France, South Africa, Colombia, and China) and are mostly associated to plasmids (Table 1). Some of these variants are sporadically accompanied by other antibiotic resistance genes like in the case of: (i) *bla*_SHV-45_ encoded by an IncA/C plasmid together with *bla*_CTX-*M*-2_ and *bla*_SHV-27_ (Dropa et al., 2015); (ii) *bla*_SHV-134_ encoded by an IncFII plasmid accompanied by a second plasmid carrying *bla*_VIM−1_ (Sánchez-Romero et al., 2012); (iii) and *bla*_SHV-105_, conferring reduced susceptibility to ceftazidime, ceftriaxone, and aztreonam together with *bla*_SHV-1_, and *bla*_SHV-5_ (Jones et al., 2009). One of the oldest variants, *bla*_SHV-6_, was only described in France in 1991 in a *K. pneumoniae* clinical case (Arlet et al., 1991). It might be speculated that the 180 kb plasmid encoding *bla*_SHV-6_ and conferring decreased susceptibility to ceftazidime and aztreonam was not stable or it reduced bacterial strain fitness preventing a successful dissemination.

Four variants have been described only in clinical *E. coli*: (i) *bla*_SHV-15_, described together with *bla*_CMY-2_ in a strain imported from India into the United Kingdom (http://www.lahey.org/studies/); (ii) *bla*_SHV-24_, identified in Japan on a transferable 150 Kb plasmid conferring high-level resistance to ceftazidime but not cefotaxime and cefazolin (Kurokawa et al., 2000); emergence of SHV-24 might have been driven by the extensive use of ceftazidime in Japan, enabling bacterial survival in high concentrations of this drug; (iii) *bla*_SHV-102_, recovered in a Spanish hospital and hydrolyzing cefotaxime and ceftazidime (Vinué et al., 2008); (iv) and *bla*_SHV-129_, detected in an abscess specimen from a patient hospitalized in Italy in 2008 (Lascols et al., 2012).

*bla*_SHV-46_ was only described on a 70 Kb conjugative plasmid also carrying *bla*_TEM-1_ and *bla*_KPC−2_ in a carbapenem-resistant strain of *Klebsiella oxytoca* from the urine of a hospitalized patient in New York (USA) in 1998 (Yigit et al., 2003). Finally, *bla*_SHV-34_ is an interesting example of extended-spectrum β-lactamase encoded by an epidemic plasmid circulating among *Citrobacter koseri, E. coli*, and *K. pneumoniae* in the same US hospital between 1998 and 2000 (Heritage et al., 2003).

Majority of SHV ESBLs have been detected in *K. pneumoniae* or *E. coli* (Table 1). *bla*_SHV-30_ was the first variant to be detected in an *E. cloacae* isolate from a blood culture from a solid-organ transplant recipient in the USA in 2003 (Szabó et al., 2005). The gene, previously described in *K. pneumoniae* and *Salmonella* (Mulvey et al., 2004; Whichard et al., 2007), was located on a 9.4 Kb plasmid and contributed together with chromosomal *ampC, bla*_SHV-7_, and *bla*_TEM-1_ to the antibiotic resistance profile of the *E. cloacae* isolate, the first of its kind producing two different SHV enzymes. Three other novel ESBL variants have been solely identified as plasmid-encoded in clinical *E. cloacae*: (i) *bla*_SHV-70_, from a Chinese patient with history of ceftazidime treatment (Ling et al., 2006) and observed in other clinical Chinese settings (Liu et al., 2008); (ii) *bla*_SHV-128_, isolated in Tunisia in 2009, located on an IncFII conjugative plasmid, and conferring resistance to all β-lactams except imipenem (Bourouis et al., 2015); (iii) and *bla*_SHV-183_, for which additional description is not available (http://www.lahey.org/studies/).

## SHV extended-spectrum β-lactamases: catalytic properties and resistance phenotype

Extended-spectrum SHV β-lactamases belong to functional group 2be, while very recently they were assigned to subclass A1 of serine β-lactamases, clustering with TEM and CTX-M enzymes among other clinically relevant β-lactamases (Bush, 2013; Philippon et al., 2016). SHV ESBLs consist of two subdomains: an α/β that includes an antiparallel five-stranded β-sheet flanked by α-helices, and an all-α-helical subdomain (Matagne et al., 1998). Similar to TEM β-lactamases (Jelsch et al., 1993), the active site is located within the cleft created by the subdomains and it contains the Ser^70^ residue that mediates the nucleophilic attack on the carbonyl group of the β-lactam ring. In the vicinity of this serine residue, several conserved structural and functional amino acid motifs have been identified. These include the Ser^70^XXLys (“SXXK” motif, with X representing variable amino acids), the Ser^130^AspAsn (“SDN” motif), the Glu^166^XXLysAsn (“EXXLN” motif), and the Lys^234^Thr/SerGly (“KTG” motif) (Bush, 2013).

Each SHV ESBL has one (SHV-2, SHV-6, SHV-8, SHV-24, SHV-27, SHV-38, SHV-41, SHV-57, SHV-98, SHV-99, SHV-102, and SHV-104) to six (SHV-128) amino acid substitutions when compared to SHV-1 (Table 2), indicating that even a single amino acid substitution is enough to convey an extended-spectrum phenotype. Therefore, we can speculate that other SHV ESBLs may still evolve from a parental SHV β-lactamase due to single spectrum-extending substitutions, although the majority of them have possibly emerged through a stepwise acquisition of several mutations (substitutions, deletions and/or insertions) from pre-existing extended-spectrum SHV variants.

**Table 2:** **Amino acid polymorphisms in SHV-type extended-spectrum β-lactamases**.

	**Amino acid position**																																														
	**7**	**8**	**10**	**14**	**18**	**20**	**25**	**35**	**43**	**54**	**61**	**64**	**75**	**80**	**89**	**96**	**97**	**104**	**122**	**123**	**129**	**140**	**142**	**146**	**148**	**154**	**156**	**163**	**169**	**179**	**186**	**187**	**188**	**192**	**193**	**195**	**202**	**205**	**238**	**240**	**243**	**271**	**274**	**275**	**276**	**282**	**286**
SHV-1	Y	I	L	S	T	P	A	L	R	G	R	E	V	V	E	H	Y	D	L	C	M	A	V	A	L	Q	G	D	L	D	M	A	A	K	L	T	R	R	G	E	A	S	E	R	N	I	L
SHV-2	.	.	.	.	.	.	.	.	.	.	.	.	.	.	.	.	.	.	.	.	.	.	.	.	.	.	.	.	.	.	.	.	.	.	.	.	.	.	S	.	.	.	.	.	.	.	.
SHV-2A	.	.	.	.	.	.	.	Q	.	.	.	.	.	.	.	.	.	.	.	.	.	.	.	.	.	.	.	.	.	.	.	.	.	.	.	.	.	.	S	.	.	.	.	.	.	.	.
SHV-3	.	.	.	.	.	.	.	.	.	.	.	.	.	.	.	.	.	.	.	.	.	.	.	.	.	.	.	.	.	.	.	.	.	.	.	.	.	L	S	.	.	.	.	.	.	.	.
SHV-4	.	.	.	.	.	.	.	.	.	.	.	.	.	.	.	.	.	.	.	.	.	.	.	.	.	.	.	.	.	.	.	.	.	.	.	.	.	L	S	K	.	.	.	.	.	.	.
SHV-5	.	.	.	.	.	.	.	.	.	.	.	.	.	.	.	.	.	.	.	.	.	.	.	.	.	.	.	.	.	.	.	.	.	.	.	.	.	.	S	K	.	.	.	.	.	.	.
SHV-6	.	.	.	.	.	.	.	.	.	.	.	.	.	.	.	.	.	.	.	.	.	.	.	.	.	.	.	.	.	A	.	.	.	.	.	.	.	.	.	.	.	.	.	.	.	.	.
SHV-7	.	F	.	.	.	.	.	.	S	.	.	.	.	.	.	.	.	.	.	.	.	.	.	.	.	.	.	.	.	.	.	.	.	.	.	.	.	.	S	K	.	.	.	.	.	.	.
SHV-8	.	.	.	.	.	.	.	.	.	.	.	.	.	.	.	.	.	.	.	.	.	.	.	.	.	.	.	.	.	N	.	.	.	.	.	.	.	.	.	.	.	.	.	.	.	.	.
SHV-9	.	.	.	.	.	.	.	.	.	Del	.	.	.	.	.	.	.	.	.	.	.	R	.	.	.	.	.	.	.	.	.	.	.	N	V	.	.	.	S	K	.	.	.	.	.	.	.
SHV-11^**^	.	.	.	.	.	.	.	Q	.	.	.	.	.	.	.	.	.	.	.	.	.	.	.	.	.	.	.	.	.	.	.	.	.	.	.	.	.	.	.	.	.	.	.	.	.	.	.
SHV-12	.	.	.	.	.	.	.	Q	.	.	.	.	.	.	.	.	.	.	.	.	.	.	.	.	.	.	.	.	.	.	.	.	.	.	.	.	.	.	S	K	.	.	.	.	.	.	.
SHV-13	.	.	.	.	.	.	.	Q	.	.	.	.	.	.	.	.	.	.	.	.	.	.	.	.	.	.	.	.	.	.	.	.	.	.	.	.	.	.	A	.	.	.	.	.	.	.	.
SHV-15	.	.	.	.	.	.	.	Q	.	.	.	.	.	M	K	.	.	.	.	.	.	.	.	.	.	.	.	.	.	.	.	.	.	.	.	.	.	.	S	K	.	.	.	.	.	.	.
SHV-16	.	.	.	.	.	.	.	.	.	.	.	.	.	.	.	T	H	.	.	.	.	.	.	.	.	.	.	Ins	.	.	.	.	.	.	.	.	.	.	.	.	.	.	.	.	.	.	.
SHV-18	.	F	.	.	.	.	.	.	S	.	.	.	.	.	.	.	.	.	.	.	.	.	.	.	.	.	.	.	.	.	.	.	.	.	.	.	.	.	A	K	.	.	.	.	.	.	.
SHV-23^*^	.	.	.	.	.	.	.	.	.	.	.	.	.	.	.	.	.	.	F	.	.	.	.	.	.	.	.	.	.	.	.	.	G	.	.	.	.	.	S	K	.	.	.	.	.	.	.
SHV-24	.	.	.	.	.	.	.	.	.	.	.	.	.	.	.	.	.	.	.	.	.	.	.	.	.	.	.	.	.	G	.	.	.	.	.	.	.	.	.	.	.	.	.	.	.	.	.
SHV-27	.	.	.	.	.	.	.	.	.	.	.	.	.	.	.	.	.	.	.	.	.	.	.	.	.	.	D	.	.	.	.	.	.	.	.	.	.	.	.	.	.	.	.	.	.	.	.
SHV-30	.	F	.	.	.	.	.	.	S	.	.	.	.	.	.	.	.	.	.	.	.	.	.	.	.	.	.	.	.	.	.	.	.	.	.	.	.	.	S	.	.	.	.	.	.	.	.
SHV-31	.	.	.	.	.	.	.	Q	.	.	.	.	.	.	.	.	.	.	.	.	.	.	.	.	.	.	.	.	.	.	.	.	.	.	.	.	.	.	.	K	.	.	.	.	.	.	.
SHV-34	.	F	.	.	.	.	.	.	S	.	.	G	.	.	.	.	.	.	.	.	.	.	.	.	.	.	.	.	.	.	.	.	.	.	.	.	.	.	S	.	.	.	.	.	.	.	.
SHV-38	.	.	.	.	.	.	.	.	.	.	.	.	.	.	.	.	.	.	.	.	.	.	.	V	.	.	.	.	.	.	.	.	.	.	.	.	.	.	.	.	.	.	.	.	.	.	.
SHV-40	.	.	.	.	.	.	.	Q	.	.	.	.	.	.	.	.	.	.	.	.	.	.	.	.	.	.	.	.	.	.	.	.	.	.	.	.	.	.	.	.	G	.	.	.	.	.	.
SHV-41	.	.	.	.	.	.	.	.	.	.	.	.	.	.	.	.	.	.	.	.	.	.	F	.	.	.	.	.	.	.	.	.	.	.	.	.	.	.	.	.	.	.	.	.	.	.	.
SHV-42	.	.	.	.	.	.	S	.	.	.	.	.	.	.	.	.	.	.	.	.	V	.	.	.	.	.	.	.	.	.	.	.	.	.	.	.	.	.	.	.	.	.	.	.	.	.	.
SHV-45	.	.	.	.	.	.	.	.	.	.	.	.	.	.	.	.	.	.	.	.	.	.	.	.	.	.	D	.	.	.	.	.	.	.	.	.	.	.	S	K	.	.	.	.	.	.	.
SHV-46	.	.	.	.	.	.	.	.	.	.	.	.	.	.	.	.	.	.	.	.	.	.	.	.	.	.	.	.	.	.	.	.	.	.	.	N	.	.	S	K	.	.	.	.	.	.	.
SHV-55	F	.	.	.	.	.	.	.	.	.	.	.	.	.	.	.	.	.	.	.	.	.	.	.	.	.	.	.	.	.	.	.	.	.	.	.	.	.	S	K	.	.	.	.	.	.	.
SHV-57	.	.	.	.	.	.	.	.	.	.	.	.	.	.	.	.	.	.	.	.	.	.	.	.	.	.	.	.	R	.	.	.	.	.	.	.	.	.	.	.	.	.	.	.	.	.	.
SHV-64	.	.	.	.	.	.	.	Q	.	.	.	.	L	.	.	.	.	.	.	.	.	.	.	.	.	.	.	.	.	.	.	.	.	.	.	.	.	.	S	K	.	.	.	.	.	.	.
SHV-66	.	.	.	.	.	.	.	Q	.	.	.	Q	.	.	.	.	.	.	.	.	.	.	.	.	.	.	.	.	.	.	.	.	.	.	.	.	.	.	S	K	.	.	.	.	.	.	.
SHV-70	.	.	.	.	.	.	.	Q	.	.	.	.	.	.	.	.	.	.	.	.	.	.	.	.	V	.	.	.	.	.	.	.	.	.	.	.	.	.	.	.	.	.	.	.	.	.	.
SHV-86	F	.	.	.	.	.	.	Q	.	.	.	.	.	.	.	.	.	.	.	.	.	.	.	.	.	.	.	.	.	.	.	.	.	.	.	.	.	.	S	R	.	.	.	.	.	.	.
SHV-90	.	.	.	.	.	.	.	.	.	.	.	.	.	.	.	.	.	.	.	.	.	.	.	.	.	.	.	.	.	.	.	T	.	.	.	.	.	.	S	K	.	.	.	.	.	.	.
SHV-91	.	.	.	.	.	S	.	.	.	.	.	.	.	.	.	.	.	.	.	.	.	.	.	.	.	.	.	.	.	.	.	.	.	.	.	.	.	.	.	K	.	.	.	.	.	.	.
SHV-98	.	.	.	.	.	.	.	.	.	.	.	.	.	.	.	.	.	.	.	.	.	.	.	.	.	.	.	.	.	.	.	.	.	.	.	.	.	.	.	.	.	I	.	.	.	.	.
SHV-99	.	.	.	.	.	.	.	.	.	.	.	.	.	.	.	.	.	G	.	.	.	.	.	.	.	.	.	.	.	.	.	.	.	.	.	.	.	.	.	.	.	.	.	.	.	.	.
SHV-100	F	.	.	.	.	.	.	Ins	.	.	.	.	.	.	.	.	.	.	.	.	.	.	.	.	.	.	.	.	.	.	.	.	.	.	.	.	.	.	.	.	.	.	.	.	.	.	.
SHV-102	.	.	.	.	.	.	.	.	.	.	.	.	.	.	.	.	.	.	.	.	.	.	.	.	.	.	.	.	.	.	.	.	.	.	.	.	.	.	A	.	.	.	.	.	.	.	.
SHV-104	.	.	.	.	.	.	.	.	.	.	.	.	.	.	.	.	.	.	.	.	.	.	.	.	.	.	.	.	.	.	.	.	.	.	.	.	S	.	.	.	.	.	.	.	.	.	.
SHV-105	.	F	.	.	.	.	.	.	S	.	.	.	.	.	.	.	.	.	.	.	.	.	.	.	.	.	D	.	.	.	.	.	.	.	.	.	.	.	S	K	.	.	.	.	.	.	.
SHV-106^*^	F	.	.	.	.	.	.	.	.	.	.	.	.	.	.	.	.	.	.	.	.	.	.	.	.	.	.	.	.	.	.	.	.	.	.	.	.	.	S	.	.	.	.	.	.	.	.
SHV-115	.	.	.	.	.	.	.	.	.	.	H	.	.	.	.	.	.	.	.	.	.	.	.	.	.	.	.	.	.	.	.	.	.	.	.	.	.	.	.	K	.	.	K	.	.	.	.
SHV-128	.	.	.	.	.	.	.	Q	.	.	.	.	.	.	.	.	.	.	.	R	.	.	.	.	.	.	.	.	.	.	.	.	.	.	.	.	.	.	S	K	.	.	.	.	.	T	P
SHV-129	.	.	.	.	.	.	.	Q	.	.	.	.	.	.	.	.	.	.	.	.	.	.	.	.	.	.	.	.	.	.	.	.	.	.	.	.	.	.	S	K	.	.	.	L	D	.	.
SHV-134	.	.	.	.	.	.	.	Q	.	.	.	.	.	.	.	.	.	.	.	.	.	.	.	.	.	E	.	.	.	.	.	.	.	.	.	.	.	.	S	K	.	.	.	.	.	.	.
SHV-183	.	.	.	.	.	.	.	Q	.	.	.	.	.	.	.	.	.	.	.	.	.	.	.	.	.	.	.	.	.	.	Ins	.	.	.	.	.	.	.	S	K	.	.	.	.	.	.	.

*Amino acid numbering is according to SHV-1 (Ambler numbering system, upper row). Dots indicate identical amino acids*.

*Amino acid positions for SHV-16 (96, 97, 163), SHV-86 (7), SHV-100 (7, 35), SHV-106 (7, 8) and SHV-183 (186) have been updated from what reported in the Lahey Clinic Website (http://www.lahey.org/studies/) according to GenBank sequences: bla_SHV-16_, AF072684.2; bla_SHV-86_, DQ328802; bla_SHV-100_, AM941846; bla_SHV-106_, AM941847; bla_SHV-183_, HG934764*.

* not confirmed as belonging to subgroup 2be;

** *SHV-11 (Subgroup 2b) is provided as reference*.

Among SHV ESBLs, amino acid substitutions are predominantly located at positions Leu^35^, Gly^238^, and Glu^240^, while other less frequent but critical substitutions for the extended-spectrum phenotype occur on several amino acids including Ile^8^, Arg^43^, Glu^64^, Gly^156^, Asp^179^, and Arg^205^ (Table 2). Although, most of these residues are not involved directly in β-lactams hydrolysis, they result in the enhancement or relaxation of the active site, enabling it to accommodate and to efficiently react with oxyimino-β-lactams (Tzouvelekis and Bonomo, 1999). Amino acid substitutions on some of these positions (Arg^43^, Asp^179^, Arg^205^, Gly^238^, and Glu^240^) have been also associated with the expansion toward an ESBL phenotype among TEM enzymes (Knox, 1995).

Residue Leu^35^ is located further away from the active site of class A β-lactamases and its substitution to Gln (e.g., SHV-2a, SHV-12) has been suggested to have an indirect role in enhancing the extended-spectrum capability of SHV β-lactamases (Nüesch-Inderbinen et al., 1997). In contrast, Gly^238^ and Glu^240^ amino acids are part of the active site lying near the R1 side chain of the β-lactam (Huletsky et al., 1993). Substitutions in Gly^238^ either to Ser (e.g., SHV-2, SHV-2a) or Ala (e.g., SHV-13, SHV-18) displace the β3-strand from the reactive Ser^70^, resulting in a slightly expanded active site. This conformational change improves the binding to and the accommodation of newer cephalosporins with large C7 substituents, thereby expanding the substrate spectrum of these SHV ESBLs to include cefotaxime and to a lesser extent to ceftazidime (Huletsky et al., 1993; Matagne et al., 1998; Nukaga et al., 2003). It has been suggested that Glu^240^ substitutions to Arg (SHV-86) or Lys (e.g., SHV-4, SHV-5) cause the ammonium group of the long side-chains of these residues to form an electrostatic bond with the carboxylic acid group on the oxyimino-substituents of ceftazidime and aztreonam (Knox, 1995). This interaction has a dual effect on the hydrolysis of ceftazidime by improving initial binding and facilitating proper positioning within the SHV β-lactamase, whereas the hydrolysis of other β-lactams is less affected (Huletsky et al., 1993). Gly^238^Ser and Glu^240^Lys amino acid substitutions characterize the majority of SHV ESBLs (Table 2) and mirror those seen in extended-spectrum TEM β-lactamases. Interestingly, a plethora of extended-spectrum SHV and TEM β-lactamases exhibit higher levels of hydrolytic activity against ceftazidime than against cefotaxime (ceftazidimases) (Table 3). This phenotype was attributed to the Glu^240^Lys substitution, in contrast with most CTX-M β-lactamases lacking this critical substitution and only showing a cefotaximase activity, (Bonnet, 2004).

**Table 3:** **Kinetic parameters of available SHV-type extended-spectrum β-lactamases**.

**Enzyme**	**Parameter**	**PEN**	**AMP**	**AMX**	**TIC**	**PIP**	**CER**	**CEF**	**CAZ**	**CTX**	**FEP**	**ATM**	**CLA**	**SUL**	**TZB**
SHV-1^**^	*K*_cat_	455		900	60	570	170	10	NH	NH	>100	NH			
	*K*_m_	20		90	22	60	110	26	ND	ND	>3000	ND			
	*K*_cat_/*K*_m_	23,000		10,000	2700	10,000	1500	400	ND	ND	>35	ND			
	*V*_max_/*K*_m_	100					4	1							
	*K*_i_												0.19	1.70	0.057
	IC_50_												0.057	7.50	0.150
SHV-2	*K*_i_												0.16	0.36	0.04
	IC50												0.020	0.57	0.049
	*V*_max_	100							6.5	70		1			
	*K*_m_	3.5	12			ND		ND	24	18	NA	10			
	*K*_cat_		206			ND		ND		11	NA				
	*K*_cat_/*K*_m_		17			ND		ND		0.6	0.008				
SHV-2a	*K*_i_		13					5	72	4		3	0.08	0.47	0.027
	IC_50_		100					53	1	10			0.018	0.68	0.038
SHV-4	*V*_max_	100							52	115		5			
	*K*_m_	3.5							60	25		0.5			
SHV-5	*K*_i_												0.10	0.18	0.036
	IC50												0.005	0.40	0.022
	*K*_m_	15	11					3	23	7		0.02			
	*V*_max_/*K*_m_	100	100					51	4	7					
SHV-7	*K*_m_							2.7	24	11		13			
	*V*_max_							35	13	30		3.3			
SHV-9	*V*_max_	100	215					58	10	24					
	*K*_m_	18	12					5	18	9					
	IC50												0.14	0.43	
SHV-13	*K*_m_	10	28			18			91	11		77			
	*V*_max_	100	178			136			0.38	12		0.66			
	*V*_max_/*K*_m_	100	64			76			0.42	11		0.86			
SHV-18	*V*_max_	100					200		13.5	26.9		< 1			
	*V*_max_/*K*_m_	100					53		1.5	24		ND			
SHV-24	*V*_max_		2				2.37		0.043			0.735			
	*K*_m_		32				210		30			500			
	*V*_max_/*K*_m_		0.0625				0.0113		0.000143			0.00147			
	*K_i_*		57				ND		37			ND			
SHV-38*^$^*	*K*_cat_	100		1800	10	100	40	5	110	1	3	3			
	*K*_m_	13		35	14	80	150	100	3800	800	1600	5500			
	*K_cat_*/*K*_m_	7700		51,000	700	1300	270	50	30	1	2	0.5			
SHV-55^*^	*K*_m_	5 ± 0.51		10 ± 0.14	6 ± 0.02	8 ± 0.37		9 ± 0.68	58 ± 7.40	21 ± 0.13	149 ± 2.61	5 ± 0.62			
	*K*_cat_	23 ± 0.76		23 ± 0.17	8 ± 0.00	27 ± 1.53		38 ± 3.94	9 ± 0.21	24 ± 0.34	30 ± 3.10	< 0.1			
	*K_cat_*/*K*_m_	5.3 ± 0.42		2.5 ± 0.002	1.5 ± 0.00	3.7 ± 0.03		4.4 ± 0.78	0.2 ± 0.02	1.1 ± 0.01	0.2 ± 0.02	ND			
	IC_50_												0.02		
SHV-57	*K*_m_	67							30.9						
	*K*_cat_	3.8 × 10^−3^							8.6 × 10^−4^						
	*K*_cat_/*K*_m_	5.67 × 10−^5^							2.78 × 10−^5^						
	*K_i_*												27 × 10^3^		1.16 × 10^3^
SHV-99^*^	*K*_m_	12 ± 0.11		11 ± 0.26	5 ± 0.93	13 ± 1.43		102 ± 11.38	136 ± 4.09	183 ± 0.72		196 ± 0.60			
	*K*_cat_	778 ± 616		563 ± 8	58 ± 2	563 ± 13		37 ± 2	< 0.1	< 0.1		0.5 ± 0.001			
	*K*_cat_/*K*_m_	62.3 ± 4.4		49.6 ± 1.8	13 ± 2.4	43.5 ± 6.5		0.37 ± 0.04	< 0.001	< 0.001		0.003			
	IC_50_												0.02		0.03
SHV-104	*K*_cat_	55			80			30		>1.8					
	*K*_m_	94			10			68		>600					
	*K*_cat_/*K*_m_	0.6			8			0.44		0.003					
SHV-129^#^^*^	*K*_cat_		22.8 ± 11			1688 ± 4		26 ± 1	3.1 ± 1.5	4.8 ± 3.4	4.5 ± 0.5				
	*Km*		46.8 ± 24			25 ± 9		12.1 ± 3.7	24 ± 3	26.7 ± 5.5	52 ± 3.5				
	*K*_cat_/*K*_m_		0.5 ± 0.7			7 ± 0.4		2.2 ± 0.3	0.13 ± 0.5	0.2 ± 0.5	0.09 ± 0.01				
	*K*_i_												0.4	0.4	0.04

*Antibiotic: Penicillin (PEN); Ampicillin (AMP); Amoxicillin (AMX); Ticarcillin (TIC); Piperacillin (PIP); Cephaloridine (CER); Cephalothin (CEF); Ceftazidime (CAZ); Cefotaxime (CTX); Cefepime (FEP); Aztreonam (ATM); Clavulanic Acid (CLA); Sulbactam (SUL); Tazobactam (TZB)*.

*Parameters are expressed as μM (K_i_, IC_50_, K_m_), s^−1^(K_cat_), and μM/min (K_cat_/K_m_, V_max_). Only antibiotics for which 3 or more SHV enzyme values were available are reported*.

*NA, not able to determine the rate of hydrolysis and affinity; NH, not hydrolyzed; ND, not determined*.

** *Non ESBL SHV-1 is provided as reference*.

$ *K_cat_/K_m_ values are expressed as mM/s*.

# *K_cat_/K_m_ values are expressed as μM/s*.

* *Values (Except IC_50_) represent mean ± standard deviation*.

*References: SHV-1 (Gutmann et al., 1989; Poirel et al., 2003); SHV-2 (Gutmann et al., 1989; Bradford et al., 1995; Winkler and Bonomo, 2016); SHV-2a (Podbielski et al., 1991); SHV-4 (Péduzzi et al., 1989); SHV-5 (Gutmann et al., 1989); SHV-7 (Bradford et al., 1995); SHV-9 (Prinarakis et al., 1996); SHV-13 (Yuan et al., 2000); SHV-18 (Rasheed et al., 2000); SHV-24 (Kurokawa et al., 2000); SHV-38 (Poirel et al., 2003); SHV-55 (Mendonça et al., 2006); SHV-57 (Ma et al., 2005): SHV-99 (Ramdani-Bouguessa et al., 2011); SHV-104 (Ben Achour et al., 2014); SHV-129 (Winkler and Bonomo, 2016)*.

Among the less frequent but critical substitutions, Ile^8^Phe in the signal sequence of the precursor of SHV ESBLs (e.g., SHV-7, SHV-18) has been associated with a more efficient β-lactamase transfer into the periplasm (Randegger et al., 2000), a proof that, beside enzymatic structure and gene expression, also the rate of transfer plays a role in resistance phenotype. On the contrary, Arg^43^Ser (e.g., SHV-7, SHV-18) and Gly^156^Asp (SHV-27, SHV-45, SHV-105) substitutions affect the structural arrangement of the conserved residues 64–69 and 166–170, respectively. These changes, opposite to the active site cavity (Ser^70^) for the hydrolysis of the β-lactam molecules, expand the active site to accommodate bulkier cephalosporins (Knox, 1995; Corkill et al., 2001). Asp^179^ amino acid is highly conserved among subclass A1 of serine β-lactamases and together with Arg^164^ form a salt bridge that links the two ends of the Ω loop. Substitutions Asp^179^Ala (SHV-6), Asp^179^Asn (SHV-8) and Asp^179^Gly (SHV-24) result in the elimination of the salt bridge with subsequent increase in ceftazidime resistance (Sowek et al., 1991). Several other amino acid substitutions (Table 2) have been described as either responsible for or possibly contributing to the ESBL phenotype, the detailed description of which exceeds the scope of this review.

Apart from point mutations leading to amino acid substitutions, frame shift mutations have been observed with very low occurrence among SHV ESBLs resulting in amino acid insertions (Arpin et al., 2001; Ramdani-Bouguessa et al., 2011) or deletions (Prinarakis et al., 1996). However, their role in the rising of the extended-spectrum phenotype remains unclear. SHV ESBL variants falling in this category are: (i) SHV-9, with the deletion of Gly^54^ (Prinarakis et al., 1996); (ii) SHV-16, with a 5-amino acid sequence duplication (Asp^163a^ArgGluTrpGluThr-Asp^163b^ArgGluTrpGluThr) of the amino acids between 163 and 167, including Glu^166^ in the Ω loop (Arpin et al., 2001); (iii) SHV-100, with a 13-amino acid insertion (SerGluSerGlnLeuSerGlyArgValGlyMetIleGlu) between amino acids 35 and 36 (Ramdani-Bouguessa et al., 2011); and (iv) SHV-183, with an Ala insertion between amino acids 186 and 187 (http://www.lahey.org/studies). Of note, the duplication observed in SHV-16 was shown to increase the conformational flexibility of the catalytic region facilitating the access of bulkier cephalosporins, such as ceftazidime, but resulted in enzymatic instability (Arpin et al., 2001). This finding could explain the low incidence of frame shift mutations among extended-spectrum SHV β-lactamases, due to a deleterious effect on the enzymes.

Overall, the available SHV ESBL kinetic parameters show that most of the substitutions lead to more efficient hydrolysis of oxyimino-β-lactams than penicillins, as depicted by the low K_*cat*_ values for penicillins (Table 3). While they retain their ability to hydrolyze penicillins, they are not catalytically so efficient compared to SHV-1 (Bush and Singer, 1989) and this is due to the decreased strength of the crucial hydrogen-bonding network needed for penicillin catalysis (turnover). As a consequence, since β-lactam inhibitors (clavulanic acid, sulbactam, and tazobactam) are structurally very similar to penicillin substrates, SHV ESBLs also exhibit increased susceptibility to β-lactam inhibitors compared to SHV-1 (Table 3) leading to less inhibitor required for inactivation (lower K_i_ and IC_50_s; Tzouvelekis and Bonomo, 1999).

## Detection

There are at least 46 known SHV-ESBL genes together with more than 150 non-ESBL or unclassified alleles to date (http://www.lahey.org/studies/). Accurate identification of these variants is essential for surveillance and for epidemiological studies of transmission mode, particularly in clinical setting, where appropriate antimicrobial therapy is critical.

A panel of different phenotypic confirmatory tests is available to determine the presence of extended-spectrum β-lactamases, including SHV-variants: minimum inhibitory concentration (MIC) determination of β-lactam with and without clavulanic acid, double disk synergy test (DDST), inhibitor potentiated disk diffusion test (IPDDT), three-dimensional test (TDT) and commercially available methods (Etest for ESBLs, Vitek ESBL cards, MicroScan panels, and BD Phoenix Automated Microbiology System (Bradford, 2001; Paterson and Bonomo, 2005). Standard microbiological procedures can take up to several days for culture, isolation and characterization and many comparative studies have shown that PCR-based methods have higher sensitivity (Bedenic et al., 2001, 2007; Singh et al., 2012), mostly due to variable levels of gene expression. Therefore, PCR and nucleotide sequence analysis (Stürenburg et al., 2003), together with various PCR-based methods, remain the gold standard for extended-spectrum β-lactamase SHV-variants identification.

Chanawong and colleagues developed a PCR-restriction fragment length polymorphism (PCR-RFLP) method to allow the identification of new SHV β-lactamases variants through detection of known mutations that alter recognition sites of restriction endonucleases (Chanawong et al., 2000). PCR-RFLP complements pre-existing PCR-single strand conformational polymorphism (PCR-SSCP) limited by partial gene amplification, thus missing potential mutation sites (M'Zali et al., 1998). PCR-RFLP can also be used in combination with restriction site insertion-PCR (RSI-PCR), a method based on primers mismatches, allowing the unambiguous identification of up to 27 SHV variants by point mutation (Chanawong et al., 2001a). Fluorescently labeled hybridization probes followed by melting curve analysis can also be used to discriminate between ESBL and non-ESBL *bla*_SHV_ genes (Randegger and Hächler, 2001). This method, termed the SHV melting curve mutation detection method, is also able to categorize SHV ESBL producers into phenotypically relevant subgroups: (i) weak ceftazidime resistance (SHV-6 and SHV-8); (ii) significant resistance to cefotaxime and ceftriaxone and moderate resistance to ceftazidime (SHV-2, SHV-2a, and SHV-3); and (iii), most effective against all expanded-spectrum cephalosporins (SHV-4, SHV-5, SHV-9, and SHV-12). Combined systems can also be developed *ad hoc* to rapidly screen local epidemiological settings (Chia et al., 2005). A modified SHV melting-curve mutation detection method able to distinguish between prevalent Taiwanese *bla*_SHV_ genes (SHV-1, SHV-2, SHV-2a, SHV-5, SHV-11, and SHV-12) was combined with a multiplex PCR to identify different β-lactamases genes (*bla*_SHV_, *bla*_CTX-*M*-3_-like, and *bla*_CTX-*M*-14_). The design of this method can be easily adapted to other geographic areas where different ESBLs are prevalent. Multiplex real-time PCR assays for the fast detection of extended-spectrum β-lactamase and carbapenemase genes were developed with differential melting curves able to recognize up to 120 different SHV allelic variants (Singh et al., 2016).

New techniques for ESBL detection are employed alongside PCR-based methods these days. Loop-mediated isothermal amplification (LAMP) was applied to detect SHV- and other ESBL-producing bacteria in meat and proved to be more specific and sensitive than MacConkey agar or cefpodoxime disc methods (Anjum et al., 2013). Commercial DNA microarrays are also proving themselves to be accurate, with sensitivity and specificity values for ESBL detection being high. Up to 53 SHV-variants can be covered on a same array (Leinberger et al., 2010), but on the other hand some alleles may fail to be detected (i.e., SHV-12), as previously reported (Stuart et al., 2012). Because arrays have major limitations to detect novel genes or variants, PCR and sequencing remains essential. Matrix-assisted laser desorption ionization-time of flight (MALDI-TOF) mass spectrometry (MS) is routinely used for bacteria identification and has been recently applied to detect ESBL-producing Enterobacteriaceae from positive blood cultures in clinical practice (Jung et al., 2014; Oviaño et al., 2014). Although, this methodology has yet to be fully validated, preliminary results show 99% sensitivity and 100% specificity, and denote a novel approach to categorize bacteria as ESBL producers.

Pyrosequencing combines standard PCR and sequencing by synthesis to rapidly determine the sequence of a target DNA region; it has been extensively used for the detection of bacterial resistance genes and bacterial community composition (Tang et al., 2016; Tian et al., 2016). This technique has been used to perform mutation analysis of *bla*_SHV_ to resolve heterogeneous sequences in clinical isolates of *K. pneumoniae* containing more than one SHV variant (Haanperä et al., 2008). An alternative protocol for pyrosequencing is the single-nucleotide polymorphism (SNP), ideal for the sequencing of mixed templates and determination of SNPs at the position of interest. This protocol has been applied to discriminate between eight *bla*_SHV_ variants from clinical isolates of *E. coli* and *K. pneumoniae*, reporting great reproducibility and ability to discriminate between sequences (Jones et al., 2009). A multiplex pyrosequencing assay coupled with qPCR amplification has also been recently developed to enable rapid and accurate detection of *bla*_SHV_ and *bla*_TEM_ -producing Enterobacteriaceae (Deccache et al., 2015). Overall, pyrosequencing can be a useful epidemiological tool for the exact identification of *bla*_SHV_ as a prerequisite for analyzing the spread of certain SHV variants.

Finally, the advent of whole genome sequencing (WGS) has taken differentiation of bacterial strains and identification of the associated antibiotic resistance gene cargo to another level. Aside from the phylogenetic analysis that WGS provides, the complete resistome of a strain can be unraveled as well as its mobilome, i.e., the mobile genetic elements that are associated with antibiotic resistance diffusion. Only this information can provide us with full understanding of complex genomic structures as observed, for example, in clinical *K. pneumoniae* genomes carrying (i) nineteen antibiotic resistance genes including *bla*_OXA-1_ and *bla*_SHV-28_ in the chromosome, *bla*_NDM-1_ in a plasmid, and *bla*_OXA-232_ in a second plasmid (Kwon et al., 2016); (ii) β-lactamase genes *bla*_KPC−2_, *bla*_SHV-11_, *bla*_TEM−169_, and *bla*_OXA-9_, together with *aac(6*′-)*Ib, aadA2*, and *aph(3*′-*)Ia* as aminoglycoside resistance encoding genes, *mph(A)* for macrolides, *oqxA* and *oqxB* for quinolone, *catA1* for phenicol, *sul1* for sulfonamide, and *dfrA12* for trimethoprim (Lee et al., 2014); or (iii) six different plasmids, adding up to 0.43 Mbp, coding for six β-lactamases (*bla*_SHV-12_, *bla*_OXA-9_, *bla*_TEM-1_, *bla*_CTX-*M*-2_, and *bla*_KPC−2_), together with *bla*_SHV-110_ and adhesin-related gene clusters on the chromosome (Perreira Ramos et al., 2014).

## Expansion toward new ecological niches

Over the last years the presence of antibiotics as well as antibiotic resistant bacteria has been shown outside the clinical environment, including water, soil and, most notably, food producing animals. When looking at SHV-variants distribution it is evident that in recent years, as for most extended-spectrum β-lactamases (Canton et al., 2012), their presence has been confirmed in virtually all ecological niches (Supplementary Table S1), making it more challenging to restrain antibiotic resistance diffusion. The most representative cases and variants will be discussed.

### Aquatic environment

In an effort to control the release of antibiotics and antibiotic resistant bacteria in the environment, aquatic environments are being investigated worldwide, whether they be natural, drinking or wastewaters. The latter are particularly worrisome given the high prevalence of *bla*_SHV_ alleles, as observed in untreated hospital wastewater in Australia (Gündogdu et al., 2013), their possible association with determinants of quinolone and other β-lactamase resistance (Calhau et al., 2015; Osinska et al., 2016), and their relatively easy transmission to surface water through waste water treatment plant discharges (Marti et al., 2013). Studies showed that SHV types, together with CTX-M and OXA genes can be significantly decreased by biological treatments such as activated sludge processing and anaerobic digestion, although not all can be effectively eliminated (Yi et al., 2015).

Urban waters are also exposed to relatively high population densities and therefore are often unprotected from biological contaminants, with people playing a crucial role in antibiotic resistance dissemination in the environment. Unusual finding of SHV-producing *Stenotrophomonas maltophilia* in a swimming recreational Serbian lake and its transient presence during summer months can be considered as a proof of its anthropogenic origin, given its nature of emerging nosocomial pathogen (Novovic et al., 2015). Similar conclusions can be drawn for SHV-producing *K. pneumoniae* and *E. cloacae* isolated from a Bangladeshi lake, which receives waste water from surrounding residents, commercial buildings and clinics in Dhaka city (Haque et al., 2014), as well as for artificial water reservoirs in Poland (Wolny-Koladka and Lenart-Boron, 2016), or urban surface waters in Malaysia (Tissera and Lee, 2013). In recent surveillance studies of different rivers and lakes in Switzerland, *bla*_SHV-12_-producing Enterobacteriaceae were isolated only in 4% of the cases (Zurfluh et al., 2013), although this variant is predominant in clinical Swiss isolates (Nüesch-Inderbinen et al., 1997). *bla*_SHV-12_ was also detected in Enterobacteriaceae from seawater, together with *tet(A)* and *sul2* in Portugal (Alves et al., 2014), and plasmid-encoded together with *bla*_TEM-1_ and/or *bla*_CTX-*M*-1_ in Croatia (Maravic et al., 2015). Finally, data on ESBL-producing Enterobacteriaceae isolated from drinking water is also increasing, reporting SHV alleles in rural water reservoirs in China (Zhang et al., 2015), or drinking water sources for First Nation communities in Canada (Fernando et al., 2016).

### Food producing animals

Food producing animals have become subject of increasing interest after several studies demonstrated that resistant strains of animal origin can be associated to human infections, possibly through the food chain (Hasman et al., 2005). Majority of SHV variants in this reservoir belong to *bla*_SHV-2_, *bla*_SHV-2*a*_, *bla*_SHV-5_, and *bla*_SHV-12_ (Supplementary Table S1) owing to their successful association with conjugative plasmids (see Section Plasmid epidemiology of *bla*_SHV-2_, *bla*_SHV-2*a*_, *bla*_SHV-5_, and *bla*_SHV-12_).

Surveillance activities in healthy animals worldwide are generating a tremendous amount of data on ESBL distribution. Most SHV β-lactamase producers are *E. coli* from swine and broiler fecal samples as observed in China (Tian et al., 2012); in Spain, with *bla*_SHV-2_ associated with *bla*_CTX-*M*-9_ and *bla*_SHV-12_ with *bla*_CTX-*M*-1_, in pigs and broilers respectively (Blanc et al., 2006); in layers, cattle, and broilers but not in swine in Japan (Hiki et al., 2013; Kameyama et al., 2013); and in the Netherlands, where healthy broilers carried *bla*_SHV-2_ in combination with *bla*_TEM-1_ or *bla*_TEM−135_ (Dierikx et al., 2010). Other Enterobacteriaceae like *K. pneumoniae* and *Citrobacter freundii* were positive for *bla*_SHV-2_ or *bla*_SHV-12_ from poultry and swine, respectively (Machado et al., 2008).

Finding ESBL producers in food producing animals is also mirrored by positive food samples worldwide, mostly retail chicken meat, as reported in Tunisia, with *E. coli* carrying *bla*_SHV-5_ isolated from different butcheries, supermarkets, and local markets (Jouini et al., 2013), or *Salmonella enterica* carrying *bla*_SHV-12_ in Japan (Noda et al., 2015). The cross-contamination between food producing animals and retail meat has been internationally demonstrated due to the detection of plasmid-borne SHV variants, such as *bla*_SHV-2_ and *bla*_SHV-2*a*_ from Canadian chicken meat and abattoir chicken cecum (Pouget et al., 2013) or *bla*_SHV-2_ and *bla*_TEM-1_ in Japan (Hiroi et al., 2011), presenting the potential for horizontal transfer between Enterobacteriaceae as a high public health concern.

SHV β-lactamase producing Enterobacteriaceae have been detected also in diseased animals, as reported for septicemic broilers due to avian pathogenic *E. coli* encoding a remarkable array of antibiotic resistance genes (*dfrA17-aadA5, bla*_TEM-1_, *bla*_CTX-*M*-15_, *bla*_OXA-1_, *bla*_SHV-2_, *tet*(A), *tet*(E), *qnrB2, aac*(6)-Ib-cr) (Ahmed et al., 2013); for *K. pneumoniae* isolated from bovine mastitis in the United Kingdom (Timofte et al., 2014) and Egypt (Ahmed and Shimamoto, 2011); and for multidrug resistant *S. enterica* serotypes Enteritidis and Typhimurium isolated from diarrheic calves (Ahmed et al., 2009).

Finally, *bla*_SHV-27_ is the only other SHV variant frequently reported as chromosomally located in *K. pneumoniae* from swine, in association with *bla*_SHV-11_ and *bla*_CTX-*M*-1_ in China (Zou et al., 2011); in *E. coli* isolated from farmed fish together with non ESBLs *bla*_SHV-1_, *bla*_SHV-11_, *bla*_SHV-25_, and *bla*_SHV-26_ (Jiang et al., 2012); and in opportunistic pathogens asymptomatically colonizing healthy milk cows (Hammad and Shimamoto, 2011).

### Wildlife, companion animals, and vegetables

ESBL diffusion has been studied extensively in Enterobacteriaceae from humans and livestock, whereas information on antibiotic resistance in the environment is still limited. Yet, the dissemination success of *bla*_SHV-12_ is confirmed by its introduction into the wildlife, notably in birds, as reported in Spain (Alcalá et al., 2015), the Netherlands (Veldman et al., 2013), Poland (Literak et al., 2010), and the Czech Republic (Dolejská et al., 2009). This success is likely associated to predominant avian clones and to efficient plasmids (Table 4, **Figure 3**) of the IncN incompatibility group, described to be more frequent in pathogenic than in commensal avian and human *E. coli* strains (Johnson et al., 2007). *bla*_SHV-5_ was also detected in *E. coli* from several birds of prey in Portugal, alone or in associations with *bla*_TEM−1b_ (Pinto et al., 2010).

**Table 4:** **Plasmid epidemiology of SHV-type extended-spectrum β-lactamases**.

**Inc Group**	Plasmid Size (Kb)^*^	***bla*_SHV_ allele^§^**	**Other Antibiotic Resistance Genes**	**Bacterial Species^#^**	**Country**	**References**
IncA/C	ND (C)	SHV-12 or SHV-2a	ND	*E. coli* (H)	Tunisia	Mnif et al., 2013
	150 (C)	SHV-12	*bla*_VIM−1_, *aac(6′)-Ib', aadA1b, catB2, sul1, dfrA14*	*A. caviae* (H)	Italy	Antonelli et al., 2016
	150 (NC)	SHV-12 (IS*26*)	*bla*_CTX-M-14_, *bla*_*DHA*−1_	*P. mirabilis* (H)	Korea	Song et al., 2011
	ND	SHV-2, SHV-5 or SHV-12	ND	*E. coli* (H)	France	Marcadé et al., 2009
	130 (C)	SHV-5 (IS*26*)	*bla*_*VEB*−1_, *bla*_VIM−1_, *aacA7, dfrA1, aadA1, bla*_OXA-1_, *bla*_TEM-1_, *aadB, arr2, cmlA5*	*P. stuartii* (H)	Greece	Giakkoupi et al., 2015
	97–145	SHV-45	*bla*_CTX-M-2_; *bla*_SHV-27_	*K. pneumoniae* (H)	Brazil	Dropa et al., 2015
	63.5–209	SHV-55	*bla*_CTX-M-2_; *bla*_SHV-28_	*K. pneumoniae* (H)	Brazil	Dropa et al., 2015
IncA/C-IncR	220 (C)	SHV-5	*bla*_VEB−1_, *bla*_VIM−1_*, rmtB, aacA7, dfrA1, aadA1*	*P. stuartii* (H)	Greece	Oikonomou et al., 2016
IncF	125	SHV-5 (IS*26*)	ND	*E. coli* (H)	Poland	Zienkiewicz et al., 2013
IncFIA-FIB	ND (C)	SHV-12	ND	*E. coli* (H)	Tunisia	Mnif et al., 2013
IncFIB	ND	SHV-12	*sul3*	*E. coli* (A)	Italy	Bortolaia et al., 2010
	95–200 (C)	SHV-2	*aadA1*	*E. coli* (A)	Canada	Pouget et al., 2013
	>23	SHV-2	ND	*K. pneumoniae* (H)	China	Wang et al., 2012
	ND	SHV-2	ND	*E. coli* (H)	France	Marcadé et al., 2009
	ND (C)	SHV-5	-	*E. coli* (H)	Uruguay	
IncFIB10	ND	SHV-12 (IS*26*)	*bla*_TEM-1_	*E. coli* (H)	UK	Doumith et al., 2012
IncFIC	ND	SHV-5	*aac(6′)-Ib', aadA1*	*S. marcescens* (H)	Uruguay	García-Fulgueiras et al., 2011
IncF-N	ND	SHV-2	*aac(6′)-Ib'*	*K. pneumoniae* (H)	Uruguay	García-Fulgueiras et al., 2011
IncFII	ND	SHV-2 or SHV-12	ND	*E. coli* (H)	France	Marcadé et al., 2009
	70–80 (C)	SHV-2a (IS*26*)	ND	*K. pneumoniae* (H)	Tunisia	Elhani et al., 2010
	100 (C)	SHV-128 (IS*26*)	ND	*E. cloacae* (H)	Tunisia	Bourouis et al., 2015
IncFII-FIA	ND (C)	SHV-12	ND	*E. coli* (H)	Tunisia	Mnif et al., 2013
IncFII-FIA-FIB	ND (C)	SHV-12	ND	*E. coli* (H)	Tunisia	Mnif et al., 2013
IncFII-FIB	ND	SHV-2	ND	*E. coli* (H)	France	Marcadé et al., 2009
	ND (C)	SHV-2a	ND	*E. coli* (H)	Tunisia	Mnif et al., 2013
IncFIIk1	200–220	SHV-2, SHV-55 or SHV-106	ND	*K. pneumoniae* (H)	Portugal	Rodrigues et al., 2014
IncFIIk5	220	SHV-55	ND	*K. pneumoniae* (H)	Portugal	Rodrigues et al., 2014
IncHI2	ND (C)	SHV-2a or SHV-12	ND	*E. coli* (H)	Tunisia	Mnif et al., 2013
	95 (C)	SHV-12 (IS*26*)	ND	*K. pneumoniae* (H)	Tunisia	Elhani et al., 2010
	310 (C)	SHV-12	*qnrB2, bla_TEM-1_*, *sul1, dfrA19, tet(D), strA, strB, aac(60)-1b*	*S*. Senftenberg (H)	Netherlands	Veldman et al., 2010
	200 (NC)	SHV-12	*tet(D)*	*S*. Concord (H)	Netherlands	Veldman et al., 2010
	290 (C)	SHV-12	*qnrB2, bla*_TEM−1_, *sul1, sul2, dfrA19, tet(D), strA, strB*,	*S*. Concord (H)	Netherlands	Veldman et al., 2010
	180, 350, 380	SHV-12	ND	*K. pneumoniae* (H)	Portugal	Rodrigues et al., 2014
	400	SHV-12	ND	*E. cloacae* (H)	Portugal	Rodrigues et al., 2014
	320 (C)	SHV-12	*qnrB2, strA/B, tet(D), clmA, sul1*	*S*. Bredeney (H)	Spain	Herrera-Leon et al., 2011
	ND (C)	SHV-12 (IS*26*)	*bla*_CTX-M-14_	*E. cloacae* (H)	Taiwan	Chen C. M. et al., 2015
	ND (C)	SHV-12 (IS*26*)	*bla*_CTX-M-3_	*E. cloacae*(H)	Taiwan	Chen C. M. et al., 2015
IncHI2 (ST1)	300 (C)	SHV-2	ND	*S*. Agona or Keurmassar (H)	Senegal	Harrois et al., 2014
IncI1	ND (C)	SHV-12	ND	*E. coli* (H)	Bulgaria	Markovska et al., 2014
	ND	SHV-12	ND	*E. coli* (H)	France	Marcadé et al., 2009
	ND	SHV-12	*sul3*	*E. coli* (A)	Italy	Bortolaia et al., 2010
	19 (C)	SHV-12	–	*E. coli* (A)	Italy	Bortolaia et al., 2011
	340 (C)	SHV-12	ND	*S*. Concord (H)	Norway (Ethiopia)	Fabre et al., 2009
	95 (C)	SHV-12	–	*E. coli* (A)	Poland	Literak et al., 2010
	10 (NC)	SHV-12	–	*S. enteritidis* (H)	Spain	de Toro et al., 2013
	60 (C)	SHV-12	*bla*_VIM−1_*-aacA4-dfrII- aadA1-catB2*	*K. pneumoniae, E. coli* (H)	Spain	Tato et al., 2007
	ND (C)	SHV-12 (IS*26*)	*bla*_CTX-M-3_	*E. cloacae* (H)	Taiwan	Chen C. M. et al., 2015
	95–200 (C)	SHV-2	*aadA1*	*E. coli, S*. Heidelberg (A)	Canada	Pouget et al., 2013
	95–200 (C)	SHV-2	–	*E. coli* (A)	Canada	Pouget et al., 2013
	95–200 (C)	SHV-2a	*aadA1, dfrA1*	*E. coli, S*. Kiambu (A)	Canada	Pouget et al., 2013
	95–200 (C)	SHV-2a	*aadA1*	*E. coli* (A)	Canada	Pouget et al., 2013
IncI1 (ST26)	ND	SHV-12	ND	*E. coli* (H)	Italy	Accogli et al., 2013
	ND	SHV-12 (IS*26*)	ND	*E. coli* (A)	Portugal	Jones-Dias et al., 2016
IncI1 (ST27, CC26)	115 (C)	SHV-2	*aadA2*	*S*. Livingstone (H)	Spain	de Toro et al., 2013
IncI1 (ST29/CC26)	ND (C)	SHV-12 (IS*26*)	ND	*E. coli* (E)	Portugal	Jones-Dias et al., 2016
IncI1 (ST3)	ND	SHV-12	ND	*E. coli* (A)	Italy	Accogli et al., 2013
	104 (C)	SHV-12	-	*E. coli* (A)	Italy	Bortolaia et al., 2011
IncK	ND (NC)	SHV-12	ND	*K. pneumoniae* (A)	England	Timofte et al., 2014
	155	SHV-2	–	*E. coli* (A)	Netherlands	Dierikx et al., 2010
IncL/M	ND (C)	SHV-12	ND	*E. coli* (H)	Tunisia	Mnif et al., 2013
	65	SHV-12	*bla*_KPC−2_, *rmtB*	*K. pneumoniae* (H)	China	Liu et al., 2015
	65	SHV-2	ND	*K. pneumoniae* (H)	Portugal	Rodrigues et al., 2014
	ND (C)	SHV-2a	ND	*E. coli* (H)	Tunisia	Mnif et al., 2013
	60–70 (C)	SHV-2a (IS*26*)	ND	*K. pneumoniae* (H)	Tunisia	Elhani et al., 2010
	ND (C)	SHV-5 (IS*26*)	*aacA4, aacC1, aadA1, sul1*	*S*. Typhimurium (H)	Italy	Villa et al., 2000
	90 (C)	SHV-5	*tet(A), aadA1, aacC1, aacA4, dfrA1*	*K. oxytoca* (H)	USA	Preston et al., 2014
IncN	ND (C)	SHV-12	ND	*E. coli* (H)	Tunisia	Mnif et al., 2013
	ND (C)	SHV-12	ND	*K. pneumoniae* (H)	Bulgaria	Markovska et al., 2014
	50	SHV-12	*bla*_VIM−1_, *qnrS*	*K. pneumoniae, E. coli* (H)	Norway	Naseer et al., 2012
	50 (C)	SHV-12	*bla*_VIM−1_, *qnrS*	*K. pneumoniae* (H)	Norway	Samuelsen et al., 2011
	>23	SHV-2 (IS*26*)	ND	*K. pneumoniae* (H)	China	Wang et al., 2012
	ND (C)	SHV-2a	ND	*E. coli* (H)	Tunisia	Mnif et al., 2013
IncN (ST1)	ND	SHV-12	*aadA2*	*E. coli* (H)	Netherlands	Dierikx et al., 2013
IncN (ST16)	50 (C)	SHV-2	ND	*S*. Miami (U)	Senegal	Harrois et al., 2014
IncP	ND (C)	SHV-12 (IS*26*)	–	*E. cloacae* (H)	Taiwan	Chen C. M. et al., 2015
	95–200 (C)	SHV-2a	*aadA1, dfrA1*	*E. coli* (A)	Canada	Pouget et al., 2013
IncX3	50 (C)	SHV-12	*bla*_KPC−2_	*K. pneumoniae* (H)	Australia	Partridge et al., 2015
	54 (C)	SHV-12	*bla*_NDM-1_	*K. pneumoniae* (H)	China	Wang et al., 2014
	54 (C)	SHV-12 (IS*26*)	*bla*_NDM-1_	*K. pneumoniae, C. freundii, E. aerogenes, E. cloacae, E. coli* (H)	China	Ho et al., 2012
	60 (C)	SHV-12	*bla*_NDM-1_, *bla*_TEM-1_	*E. coli* (H)	China	Huang et al., 2016
	60 (C)	SHV-12	*bla*_NDM-1_	*E. coli* (H)	China	Huang et al., 2016
	54 (C)	SHV-12 (IS*26*)	*bla*_NDM-1_	*E. coli* (H)	China	Feng et al., 2015
	54 (C)	SHV-12 (IS*26*)	*bla*_NDM−1_	*C. freundii* (H)	China	Du et al., 2013
	50	SHV-12	*qnrB7*	*E. coli* (A)	Czech Republic	Dobiasova and Dolejska, 2016
	40	SHV-12	*qnrS1*	*E. coli* (E)	Czech Republic	Dobiasova and Dolejska, 2016
	53	SHV-12 (IS*26*)	*bla*_KPC−2_	*K. pneumoniae* (H)	France	Kassis-Chikhani et al., 2013
	50 (C)	SHV-12	*bla*_NDM-1_	*E. cloacae* (H)	UAE	Sonnevend et al., 2013
	50 (C)	SHV-12	*bla*_NDM-1_	*E. coli* (H)	UAE	Sonnevend et al., 2013
	50 (C)	SHV-12	*bla*_NDM-1_	*C. freundii* (H)	UAE	Sonnevend et al., 2013
	43 (C)	SHV-12 (IS*26*)	-	*E. cloacae* (H)	USA	Hargreaves et al., 2015
IncX3-N	80	SHV-12	*bla*_TEM-1_, *qnrS1*	*E. coli* (A)	Germany	Dobiasova and Dolejska, 2016
ColETp	10 (NC)	SHV-12	*qnrS1*	*S*. Typhimurium (H)	Spain	Herrera-Leon et al., 2011
R	70	SHV-12	ND	*K. pneumoniae* (H)	Portugal	Rodrigues et al., 2014
R+IncFIIk1	300	SHV-2	ND	*K. pneumoniae* (H)	Portugal	Rodrigues et al., 2014
Untypable	90–140 (C)	SHV-12 (IS*26*)	ND	*K. pneumoniae* (H)	Tunisia	Elhani et al., 2010
	ND	SHV-12 (IS*26*)	-	*E. coli* (H)	UK	Doumith et al., 2012
	ND	SHV-12 (IS*26*)	*bla*_TEM−1_	*E. coli* (H)	UK	Doumith et al., 2012
	ND	SHV-12 (IS*26*)	*bla*_TEM-1_, *bla*_*OXA*-1_, *qnrS1*	*E. coli* (H)	UK	Doumith et al., 2012
	50 (C)	SHV-12	*bla*_NDM-1_	*K. pneumoniae* (H)	UAE	Sonnevend et al., 2013

* *C, conjugative; NC, non-conjugative; when blank is because not determined*.

§ *When present, IS26 is indicated in parenthesis*.

# *H, human; A, animal (mostly poultry, turkey and broilers; check reference for full description); E, environment. ND, not determined*.

Emergence of Enterobacteriaceae producing β-lactamases in companion animals have been gradually reported, with CTX-M enzymes being prevalent as observed in the human scenario (Rubin and Pitout, 2014). Few studies, on both healthy and diagnostic clinical canine and feline samples, report finding other ESBL variants including *bla*_SHV-3_ in the USA (Shaheen et al., 2011), *bla*_SHV-2_ in Mexico (Rocha-Gracia et al., 2015), *bla*_SHV-12_ in Italy and Poland (Carattoli et al., 2005b; Rzewuska et al., 2015) and *bla*_SHV-12_ in association with *bla*_OXA-48_, *bla*_CMY-2_, *bla*_TEM-1_, *aac(6*′*)-Ib-cr*, and *qnrB2* in Germany (Stolle et al., 2013).

Lastly, SHV variants have been detected in imported vegetables in Switzerland together with *bla*_SHV-12_ for the first time in the opportunistic foodborne pathogen *Cronobacter sakazakii* whose potential to cause bacteremia and meningitis is an actual concern (Zurfluh et al., 2015). Similar results were observed in vegetables collected in South Korea (Kim et al., 2015), salads in the Netherlands (Reuland et al., 2014), and Spain (Egea et al., 2011), displaying a new route of introduction for ESBLs and pathogenic Enterobacteriaceae.

## Plasmid epidemiology of *bla*_SHV−2_, *bla*_SHV−2a_, *bla*_SHV−5_, and *bla*_SHV−12_

**Figure 2:**
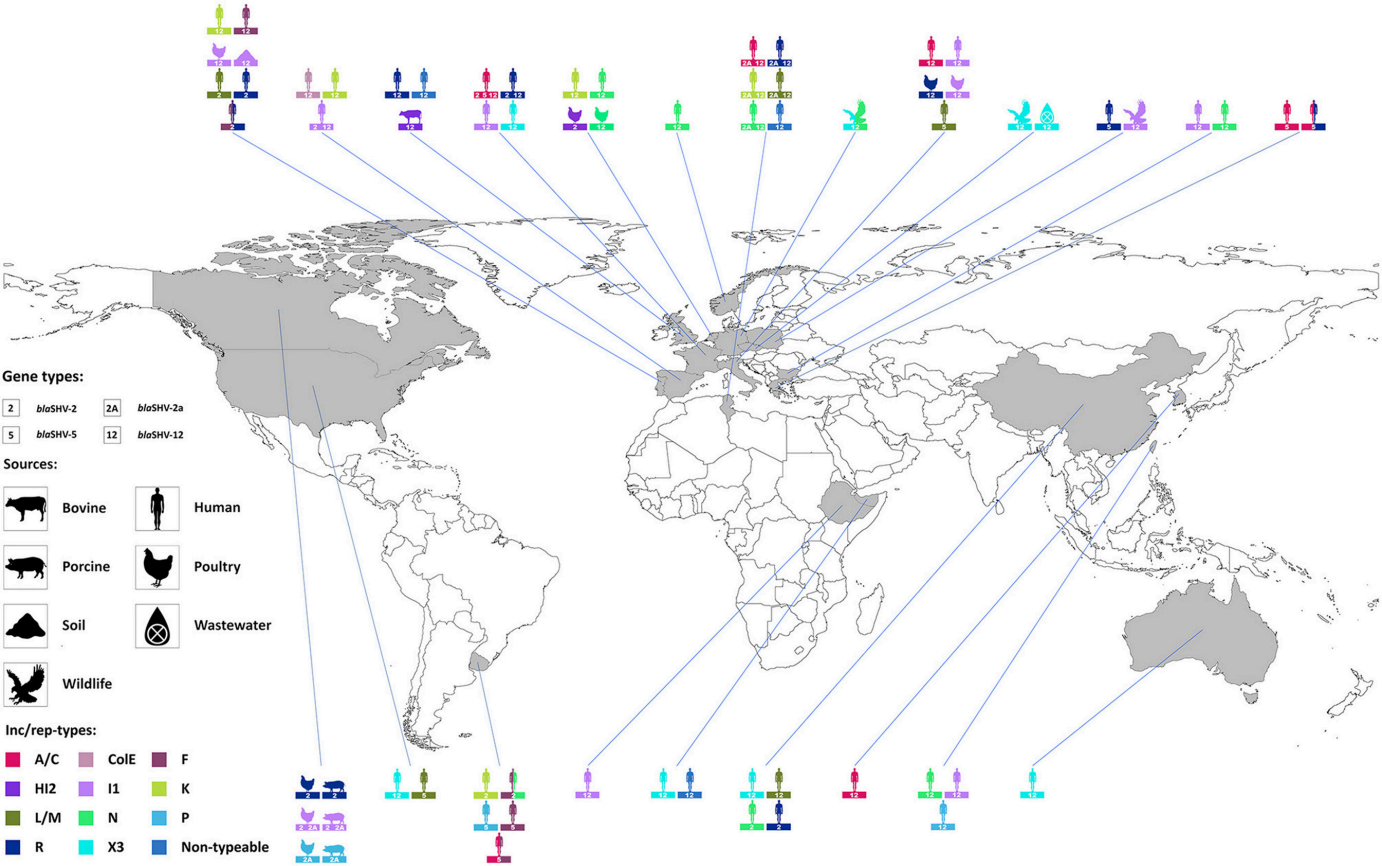
**Worldwide distribution of plasmid families encoding *bla*_SHV-2_, *bla*_SHV-2a_, *bla*_SHV-5_, and *bla*_SHV-12_**. Inc/rep types are represented in different colors; diverse symbols depict human, bovine, porcine, poultry, wildlife, soil, or wastewater sources. For a complete full reference list see Table 4.

The role of plasmids in the successful spread of β–lactamase genes has been extensively described (Carattoli, 2009, 2013) and, among the SHV family, it finds its best examples in *bla*_SHV-2_, *bla*_SHV-2*a*_, *bla*_SHV-5_, and *bla*_SHV-12_. Combination of these alleles with different dissemination machineries has brought the enzymes to reach diverse niches worldwide (Figure 2). Plasmids belonging to seven replicon types (A/C, F, HI2, I1, L/M, N, X3) have been shown to drive the epidemiology of these four predominant SHV ESBLs, although their distribution varies on the plasmid families (Table 4). Other rep families that have been only incidentally associated with extended-spectrum SHV β–lactamases include the ColE, K, P, and R (Table 4).

### IncA/C

*bla*_SHV-12_ has been identified on mostly conjugative broad-host range IncA/C plasmids in a variety of bacterial species, including *E. coli, Proteus mirabilis* and *Aeromonas caviae*, isolated from clinical samples in Tunisia, France, Korea and Italy (Marcadé et al., 2009; Song et al., 2011; Mnif et al., 2013; Antonelli et al., 2016). *E. coli* isolates recovered from clinical specimens encoding *bla*_SHV-2_, *bla*_SHV-2*a*_, and *bla*_SHV-5_ have been also identified in Tunisia and France (Marcadé et al., 2009; Mnif et al., 2013), whereas *Providencia stuartii* isolates encoding *bla*_SHV-5_ on either IncA/C or multireplicon IncA/C-R plasmids have been reported from different outbreaks in Greece (Giakkoupi et al., 2015; Oikonomou et al., 2016). Interestingly, these IncA/C plasmids (130–220 Kb) often carried multiple resistance genes, conferring multidrug resistant phenotypes (Giakkoupi et al., 2015; Antonelli et al., 2016; Oikonomou et al., 2016), resulting in the proliferation of the SHV ESBLs by co-selection.

### IncF

Plasmids belonging to the narrow-host range IncF group, including plasmids with fused replicons, have been reported to accommodate *bla*_SHV-12_ among clinical *E. coli* isolates from France (IncFII), Tunisia (IncFIA-FIB, IncFII-FIA, IncFII-FIA-FIB) and United Kingdom (IncFIB), but also among food-producing animals from Italy (IncFIB) (Marcadé et al., 2009; Bortolaia et al., 2010; Doumith et al., 2012; Mnif et al., 2013). IncF plasmids account for the dissemination of *bla*_SHV-2_ gene among *E. coli* from both clinical specimens in France (IncFIB, IncFII, IncFII-FIB) and food-producing animals (avian and porcine sources) in Canada (IncFIB), as well as in clinical *K. pneumoniae* isolates belonging to ST654 and ST15 from China (IncFIB) and Portugal (IncFII), respectively (Marcadé et al., 2009; Wang et al., 2012; Pouget et al., 2013; Rodrigues et al., 2014). Finally, clinical *E. coli* and *K. pneumoniae* from Tunisia were found to encode *bla*_SHV-2*a*_ (Elhani et al., 2010; Mnif et al., 2013), clinical *E. coli* from Poland encoded *bla*_SHV-5_ on IncF plasmids, as well as clinical *K. pneumoniae* and *Serratia marcescens* from Uruguay (García-Fulgueiras et al., 2011; Zienkiewicz et al., 2013), whereas the same plasmids have been associated with less prevalent SHV ESBLs (*bla*_SHV-55_ and *bla*_SHV-106_) in clinical *K. pneumoniae* isolates from Portugal (Rodrigues et al., 2014).

### IncHI2

In contrast with the IncA/C and IncF plasmids, the broad-host range IncHI2 group is responsible mainly for the dissemination of *bla*_SHV-12_, although this group has been found incidentally to also accommodate *bla*_SHV-2*a*_ (Mnif et al., 2013). Plasmids of this group varying in sizes (95–400 Kb) have been reported to encode *bla*_SHV-12_ in various bacterial species, such as *E. coli, K. pneumoniae, E. cloacae*, and at least three *S. enterica* serotypes (Bredeney, Concord, and Senftenberg) from human specimens with diverse geographical origin (Netherlands, Portugal, Spain, Taiwan, Tunisia; Elhani et al., 2010; Veldman et al., 2010; Herrera-Leon et al., 2011; Mnif et al., 2013; Rodrigues et al., 2014; Chen C. M. et al., 2015). Apart from *bla*_SHV-12_, some of these conjugative plasmids have been reported to co-encode for other resistance genes, including additional SHV ESBLs (*bla*_CTX-*M*-3_, *bla*_CTX-*M*-14_; Veldman et al., 2010; Chen C. M. et al., 2015).

### IncI1

The IncI1 group, consisting of narrow-host range mostly conjugative plasmids, ranks amongst the top facilitators of *bla*_SHV-2_, *bla*_SHV-2*a*_, and *bla*_SHV-12_ genes. The range of bacterial species they have encountered is limited to *E. coli, K. pneumoniae, E. cloacae*, and the *S. enterica* serotypes Concord, Enteritidis, Heidelberg and Kiambu. Nevertheless, IncI1 plasmids (19–340 Kb) occur in very diverse settings: *bla*_SHV-2_- and *bla*_SHV-12_-encoding isolates from human infections (Bulgaria, France, Italy, Spain, Taiwan; Tato et al., 2007; Marcadé et al., 2009; Accogli et al., 2013; de Toro et al., 2013; Markovska et al., 2014; Chen C. M. et al., 2015) and colonization (Ethiopia) (Fabre et al., 2009); *bla*_SHV-2_-, *bla*_SHV-2*a*_–, and *bla*_SHV-12_-encoding isolates from poultry (Canada, Italy, Portugal; Bortolaia et al., 2010, 2011; Accogli et al., 2013; Pouget et al., 2013; Jones-Dias et al., 2015), *bla*_SHV-2_- and *bla*_SHV-2*a*_-encoding isolates from pigs (Canada) (Pouget et al., 2013); *bla*_SHV-12_-encoding isolates from aquatic birds (Poland) (Literak et al., 2010); and *bla*_SHV-12_-encoding isolates from farming soil (Portugal) (Jones-Dias et al., 2016). Remarkably, *bla*_SHV-12_ on IncI1 plasmids belonging to pST26 have been identified among *E. coli* isolates of human and animal origin (Accogli et al., 2013; Jones-Dias et al., 2015), indicating the potential transmission of these *bla*_SHV-12_-encoding vehicles from human to animals and/or vice versa.

### IncL/M and IncN

The broad-host range IncL/M and IncN plasmids contribute to a lesser extent to the epidemiology of *bla*_SHV-2_, *bla*_SHV-2*a*_, *bla*_SHV-5_, and *bla*_SHV-12_ than the above-mentioned families. IncL/M plasmids (60–90 Kb) carrying SHV ESBL genes have been reported only among *E. coli, K. pneumoniae, K. oxytoca*, and *S. enterica* serotype Typhimurium of human origin in Portugal (*bla*_SHV-2_), Tunisia (*bla*_SHV-2*a*_, *bla*_SHV-12_), Italy (*bla*_SHV-5_), USA (*bla*_SHV-5_), and recently in China (*bla*_SHV-12_) (Villa et al., 2000; Elhani et al., 2010; Mnif et al., 2013; Preston et al., 2014; Rodrigues et al., 2014; Liu et al., 2015). The same bacterial species mostly from human sources carry IncN plasmids (~50 Kb) encoding *bla*_SHV-2_ (China, Senegal), *bla*_SHV-2*a*_ (Tunisia) or *bla*_SHV-12_ (Bulgaria, Netherlands, Norway, Tunisia; Samuelsen et al., 2011; Naseer et al., 2012; Wang et al., 2012; Dierikx et al., 2013; Mnif et al., 2013; Harrois et al., 2014; Markovska et al., 2014). Interestingly, the presence of IncN (pST1) plasmids encoding *bla*_SHV-12_ has been reported among *E. coli* from human and animal sources (Dierikx et al., 2013), mirroring the situation for IncI1 plasmids and underscoring the contribution of this plasmid family in the transmission of *bla*_SHV-12_ within or between these niches.

### IncX3

The IncX3 plasmid subgroup consists of narrow-host range plasmids and plays an important role in the exclusive dissemination of *bla*_SHV-12_. Conjugative plasmids (40–60 Kb) of this subgroup have been identified in diverse bacterial species (*E. coli, K. pneumoniae, C. freundii, E. aerogenes, E. cloacae*), sources (human, animal, environment) and geographical areas (Australia, China, Czech Republic, France, United Arab Emirates, US; Ho et al., 2012; Du et al., 2013; Kassis-Chikhani et al., 2013; Sonnevend et al., 2013; Wang et al., 2014; Feng et al., 2015; Hargreaves et al., 2015; Partridge et al., 2015; Dobiasova and Dolejska, 2016; Huang et al., 2016). Interestingly, the majority of these plasmids appear to co-harbor carbapenemase genes (*bla*_KPC-2_, *bla*_NDM-1_), whereas the co-localization of SHV ESBL and carbapenemase genes was reported only on IncA/C or IncA/C-R (*bla*_VIM−1_), IncL/M (*bla*_KPC−2_), and IncN (*bla*_VIM−1_) plasmids (Samuelsen et al., 2011; Naseer et al., 2012; Giakkoupi et al., 2015; Oikonomou et al., 2016), enhancing the plasmid potential maintenance among bacterial populations and the subsequent preservation and dissemination of the SHV ESBL genes.

### Miscellaneous plasmids

*bla*_SHV-12_ has been incidentally found on: (i) a ColE plasmid from *S. enterica* serotype Typhimurium DT104b in Spain (Herrera-Leon et al., 2011); (ii) an IncK plasmid from *K. pneumoniae* in the United Kingdom (Timofte et al., 2014); (iii) an IncP plasmid from *E. cloacae* in Taiwan (Chen C. M. et al., 2015); and (iv) a plasmid assigned to the R replicon type from *K. pneumoniae* in Portugal (Rodrigues et al., 2014). *E. coli* and *K. pneumoniae* encoding *bla*_SHV-2_ on IncK plasmids were recovered from animal and human sources in the Netherlands and in Uruguay, respectively (Dierikx et al., 2010; García-Fulgueiras et al., 2011). IncP plasmids encoding *bla*_SHV-2*a*_ from animals in Canada and *bla*_SHV-5_ from human in Uruguay have also been reported (García-Fulgueiras et al., 2011; Pouget et al., 2013). Finally, a number of reports highlight the presence of *bla*_SHV-12_ on mostly conjugative non-typeable plasmids, according to the PCR-based replicon-typing scheme (Carattoli et al., 2005a). These plasmids of human origin, varying between 50 and 140 Kb in size, were mostly detected among *E. coli* from the United Kingdom (Doumith et al., 2012) and *K. pneumoniae* from Tunisia (Elhani et al., 2010) and United Arab Emirates (Sonnevend et al., 2013), underscoring that their dissemination is wider than we know.

### IS26 role in *bla*_SHV_ mobilization

**Figure 3:**
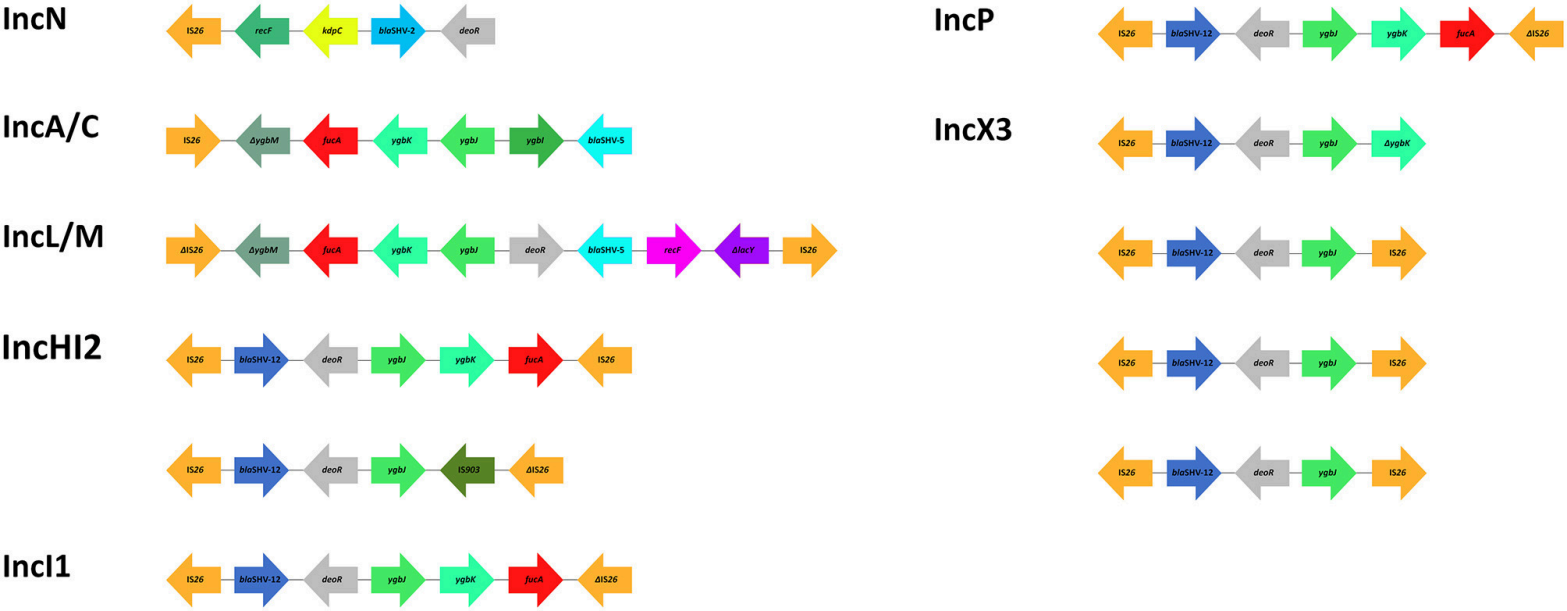
**Schematic representation of *bla*_SHV_ genetic surroundings and IS*26* association**. Common occurring features are color coded. Map is not to scale. References: IncN (Wang et al., 2012); IncA/C (Giakkoupi et al., 2015); IncL/M (Preston et al., 2014); IncHI2, IncI1, IncP (Chen C. M. et al., 2015); IncX3 (Ho et al., 2012; Du et al., 2013; Kassis-Chikhani et al., 2013; Feng et al., 2015).

Analysis of the sequences bracketing several SHV ESBL genes (*bla*_SHV-2_, *bla*_SHV-2*a*_, *bla*_SHV-5_, *bla*_SHV-12_, *bla*_SHV-106_, and *bla*_SHV-134_) among Gram-negative bacteria, including Enterobacteriaceae and non-fermenters, revealed that these β-lactamase genes are mostly associated with the IS*26* element (Table 4, Supplementary Table S1). Beside SHV ESBLs, this member of the IS*6* insertion sequence family (Mahillon and Chandler, 1998), has been associated with a plethora of resistance genes (Allard et al., 1993; Miriagou et al., 2005; Post and Hall, 2009; Cain et al., 2010; Hordijk et al., 2011) and has been found to contribute to their expression by supplying a promoter -35 box that can be coupled with a -10 box in the adjacent DNA (Lee et al., 1990; Cain and Hall, 2011). In contrast to other insertions sequences, it has been suggested that IS*26* transposes preferentially within plasmids rather than into the chromosome (He et al., 2015), possibly explaining the linkage of IS*26* and the four predominant SHV ESBL genes with IncA/C (*bla*_SHV-5_, *bla*_SHV-12_), IncF (*bla*_SHV-2*a*_, *bla*_SHV-5_, *bla*_SHV-12_), IncHI2 (*bla*_SHV-12_), IncI1 (*bla*_SHV-12_), IncL/M (*bla*_SHV-2*a*_, *bla*_SHV-5_), IncN (*bla*_SHV-2_), IncP (*bla*_SHV-12_), IncX3 (*bla*_SHV-12_), and non-typeable (*bla*_SHV-12_) plasmids (Table 4). Similarly to most antibiotic resistance genes, IS*26*-mediated mobilization of SHV ESBL genes on conjugative plasmids facilitated their subsequent intra- and inter-species dissemination (Table 4). Available sequences of transposons flanked by copies of intact and/or truncated IS*26* elements (Figure 3) and coding for SHV ESBL genes show the presence of other co-linear genes originating from the chromosome of *K. pneumoniae* (i.e., *fucA, ygbI, ygbK, ygbJ, ygbM, deoR*; Ho et al., 2012; Wang et al., 2012; Du et al., 2013; Kassis-Chikhani et al., 2013; Preston et al., 2014; Chen C. M. et al., 2015; Feng et al., 2015; Giakkoupi et al., 2015), likely underscoring the involvement of IS*26* in the mobilization of *bla*_SHV_ from the chromosome of *K. pneumoniae*, as previously suggested (Haeggman et al., 1997).

## Outside of the enterobacteriaceae and a few peculiar SHV ESBLs

SHV β–lactamases have virtually invaded all human, environmental and animal sceneries, mostly associated to Enterobacteriaceae. In recent years, the first reports of alternative bacterial hosts have been described, notably in Aeromonads, ubiquitous in aquatic habitats and occasionally able to cause human infections. *bla*_SHV-12_ was detected, in association with *bla*_FOX-2_ and *bla*_CTX-*M*-15_, on the chromosome of the foodborne pathogens *A. caviae* and *Aeromonas hydrophila* from wild-growing mussels from Croatia (Maravić et al., 2013). The first identification of plasmid-encoded SHV-12, together with VIM-1, occurred in clinical *A. caviae* accountable for a newborn bloodstream infection (Antonelli et al., 2016). The coproduction of these enzymes highlights the potential risks for public health and the role of Aeromonads as reservoirs and dissemination tools of resistance determinants in both environmental and clinical settings.

Occasionally, SHV ESBL-producing *Pseudomonas aeruginosa* can be detected in nosocomial settings and can pose a serious threat as healthcare-associated infection in many regions of the world. *bla*_SHV-2*a*_ was first identified on the chromosome of a 1995 clinical *P. aeruginosa* strain, with high sequence homology to plasmid pMPA2a from *K. pneumoniae* indicating a likely enterobacterial gene origin (Naas et al., 1999). Subsequent studies demonstrated the insertion of *bla*_SHV_ alleles into *P. aeruginosa* chromosome: *bla*_SHV_ in China (Chen Z. et al., 2015) and Iran (Shahcheraghi et al., 2009); *bla*_SHV-5_ (Poirel et al., 2004) and *bla*_SHV-12_ (Neonakis et al., 2003) in Greece; and *bla*_SHV-2*a*_ in Tunisia (Mansour et al., 2009) and France (Hocquet et al., 2010; Jeannot et al., 2013). The role of IS26 in the mobilization of *bla*_SHV-12_ was demonstrated by the chromosomal insertion of an IS26 composite transposon (>24 kb) thanks to the co-mobilization of antibiotic resistance *aac(6*′*)-Ib*, which confers amikacin resistance, likely occurred during the clinical course of a burn infection, immediately after amikacin administration (Uemura et al., 2010). *bla*_SHV-5_, *bla*_SHV-11_, *bla*_SHV-12_ were also detected in different combinations, together with *bla*_TEM-1*b*_, on various plasmids in Thailand (Chanawong et al., 2001b).

Finally, one of the most effective associations outside of the Enterobacteriaceae is with *Acinetobacter baumannii*, contributing to the worrisome spread of ESBL-producing strains especially in clinical outbreaks (Blackwell et al., 2016). Plasmid transfer from nosocomial SHV-encoding Enterobacteriaceae seems to be responsible for this phenomenon, as observed for SHV-12 in the Netherlands (Naiemi et al., 2005), or SHV-5 in the USA, a country where this variant is the most prevalent ESBL gene in Enterobacteriaceae (Naas et al., 2007).

Among all ESBL SHV β-lactamases, few enzymes deserve special consideration because of their unique enzymatic features.

SHV-38 is a unique allelic variant of the SHV family to have an expanded-spectrum to carbapenems. It was first described in *K. pneumoniae* from France (Poirel et al., 2003) and it holds a point mutation (Ala^146^Val) compared to the chromosome-encoded SHV-1. Among all 46 available SHV ESBL variants, only SHV-38 possess the Ala^146^Val substitution (Table 2), likely inducing subtle structural conformational changes favoring imipenem but not meropenem hydrolysis (Walther-Rasmussen and Høiby, 2007).

SHV-129 is a novel clinically acquired variant identified in 2012 from an Italian *E. coli* isolate (Table 1; Lascols et al., 2012) and it represents an interesting example of enzyme evolution due to antibiotic pressure. Alongside two well-known amino acid substitutions (Gly^238^Ser, Glu^240^Lys), SHV-129 contains new substitutions, Arg^275^Leu and Asn^146^Asp). The latter was recently demonstrated to be the first global suppressor substitution identified in the SHV β-lactamase family (Winkler and Bonomo, 2016), likely helping in protein stabilization and functionality, as well as in the ability of the enzyme to acquire additional substitutions. It is also proposed that due to the increasing clinical use of cefepime, SHV-129 might have evolved from SHV-2 or SHV-5 in an alternative conformation to expand its spectrum to hydrolyze cefepime, as mirrored by the kinetic parameters of the three enzymes (Table 3).

Finally, SHV-2 can be located on both chromosome and self-transmissible plasmids (Table 4; Supplementary Table S1). Association of *bla*_SHV-2_ with RCS47, a P1-like bacteriophage that infects and lysogenizes *E. coli* and several other enteric bacteria, was recently reported (Billard-Pomares et al., 2014). *bla*_SHV-2_ is flanked by two IS*26* elements that likely drove the insertion in the phage backbone. The P1-like prophages were found with high prevalence in natural *E. coli* of both animal and human origin, including ESBL-producing isolates. This kind of association was already reported for other β-lactamases (*bla*_TEM_, *bla*_CTX-*M*_, and *mecA*) from river and urban sewage water (von Wintersdorff et al., 2016), suggesting that bacteriophages might play a wider role in favoring horizontal transfer of antibiotic resistance determinants than initially thought (Muniesa et al., 2013).

## Concluding remarks

Tzouvelekis and Bonomo suggested than “it will not be surprising if (SHV) enzymes will continue to expand their substrate spectrum as long as the current antibiotics, or novel ones derived from the basic β-lactam structure, are used” (Tzouvelekis and Bonomo, 1999). In the last two decades we observed the appearance of multiple SHV-type variants, with few ones significantly expanding their substrate. One exception is represented by SHV-38, the only known SHV allelic variant able to hydrolyze carbapenems (Poirel et al., 2003), a feature that has not been associated with any TEM or CTX-M enzyme. In this image resides the fate of SHV extended β-lactamases, unable to undergo the dominant propagation observed, for instance, for the CTX-M family but yet contributing to β-lactam resistance in a not negligible way.

The persistence of SHV enzymes in the bacterial community might also be secured by co-selection with emerging resistance genes. Association of *bla*_SHV-12_ with IncX3 plasmids carrying carbapenemase genes *bla*_KPC−2_ and *bla*_NDM_ has been observed in recent years (Table 4) and it seems to be a phenomenon occurring in clinical carbapenem-resistant Enterobacteriaceae worldwide (Kassis-Chikhani et al., 2013; Sonnevend et al., 2013; Partridge et al., 2015; Huang et al., 2016). As highlighted in this review, the association of successful variants *bla*_SHV-2_, *bla*_SHV-2*a*_, *bla*_SHV-5_, and *bla*_SHV-12_ with different families of conjugative plasmids (IncA/C, IncF, IncHI2) might also underlie the colonization of virtually all ecological niches encompassing food producing animals, aquatic environment, wildlife, companion animals, and vegetables. Plasmid mediated transfer from nosocomial Enterobacteriaceae enabled SHV dispersion toward alternative bacterial hosts such as the emerging nosocomial pathogens of aquatic origin *S. maltophilia* and *A. caviae*, or contributed to the worrisome spread of ESBL-producing strains of *A. baumannii* and *P. aeruginosa*. Most interestingly, the ubiquitous presence of SHV ESBL genes and plasmids is suggestive for transmission in human, animals, and the environment, most likely through the food chain, highlighting the potential risks for public health and endorsing a one health research approach.

Overall, SHV ESBL enzymes have kept a stable role in antibiotic resistance over the years. Allele diversification is still occurring, the latest variant being identified in *E. cloacae* in 2014 (*bla*_SHV-183_), and effective associations with new genetic platforms are taking place helping expansion toward novel bacterial hosts and reservoirs.

## Author contributions

The paper was written by AL and DC, and reviewed by DM. All authors discussed, read, contributed to and approved the final manuscript.

### Conflict of interest statement

The authors declare that the research was conducted in the absence of any commercial or financial relationships that could be construed as a potential conflict of interest.

## Footnotes

^1^Of the 194 variants available on line (http://www.lahey.org/studies), 5 were withdrawn or invalidated as only partial sequence.
