# Supplementary material for: A Review of SHV Extended-Spectrum β-Lactamases: Neglected Yet Ubiquitous
Source: Front Microbiol. 2016 Sep 5;7:1374. doi: 10.3389/fmicb.2016.01374 (PMC5011133; doi:10.3389/fmicb.2016.01374)
Supplement: Supplementary file 1 [file Table1.PDF]

Supplementary Table S1. SHV-type extended-spectrum β-lactamase producing bacteria of human, animal or environmental origin, their geographical distribution, year of isolation and genetic background.

Gene	Accession Number	pI	Location	Year*	Bacterial Species[§]	Genetic Location[¥]	Conjugative Plasmid	Plasmid Size (Kb)	Other Antibiotic Resistance Genes	Reference[#]
bla$_{SHV-2}$	AF148851	7.6	Germany	1983	*K. ozaenae*	pBP60	Yes	45	ND	(Kliebe *et al.*, 1985)
			Spain	2003-2004	*E. coli*	ND	ND	ND	ND	(Vinue *et al.*, 2008)
			Vietnam	2000-2001	*E. coli*; *K. pneumoniae*	ND	ND	ND	ND	(Cao *et al.*, 2002)
			Portugal	2003	*K. pneumoniae*	ND	ND	ND	ND	(Machado *et al.*, 2007)
			Canada	1999-2000	*K. pneumoniae*	P	Yes	Variable	ND	(Mulvey *et al.*, 2004)
			Thailand	2004-2005	*K. pneumoniae*	P	Yes	ND	CTX-M-14; TEM-1	(Kiratisin *et al.*, 2008)
			France	2002	*E. coli*	Phage (IS*26*, IS*5*)	-	-	-	(Billard-Pomares *et al.*, 2014)
			Senegal	1990	*Salmonella* Miami; *E. coli*	IncN	Yes	50	-	(Harrois *et al.*, 2014)
			Uruguay	2009	*E. coli*	IncN; IncFIC; IncF	No	ND	-	(García-Fulgueiras *et al.*, 2011)
			South Africa	2001	*Salmonella* Isangi and Saint-paul	ND	ND	ND	TEM-1; CMY-2	(Usha *et al.*, 2008)
			Bulgaria	1999-2000	*K. pneumoniae; S. marcescens;S. enterica* Corvallis	P	Yes	ND	ND	(Markovska *et al.*, 2008)
			USA	2003-2004	*E. cloacae*	P	ND	ND	-	(Szabó *et al.*, 2005a)
			France	2001-2003	*K. oxytoca*	P	Yes	ND	TEM-1; OXY-1	(Decré *et al.*, 2004)
			Indonesia	2001-2002	*E. coli; K. pneumoniae*	ND	ND	ND	CTX-M-9 or TEM-1	(Severin *et al.*, 2012)
			Spain	2001-2005	*S. enterica* Livingstone	IncI1	Yes	125	aadA1;	(González-Sanz *et al.*, 2009)
			Mexico	1990-1996	*K. pneumoniae; K. variicola*	P	Yes	Variable	ND	(Garza-Ramos *et al.*, 2007)
			Bolivia, Peru	2005	*E. coli*	C	-	-	TEM-1	(Pallecchi *et al.*, 2007)
			Taiwan	NA	*K. pneumoniae*	pK245	Yes	98	*aacC2; strA; strB; catA2; sul2; tetD; dfrA14; qnrS*	(Chen *et al.*, 2006)
			Turkey	NA	*E. coli; K. pneumoniae*	P	Yes	ND	-	(Tasli and Bahar, 2005)
			Argentina	1998	*S. flexneri*	P	Yes	ND	ND	(Andres *et al.*, 2005)
			China	2006-2009	*E. coli* (A)	ND	ND	ND	TEM-1	(Tian *et al.*, 2012)
			Canada	2006-2007	*E. coli* (E)*; S. enterica* (A)	IncI1; IncFIB	-	-	*tetA/B; sul1; dfrA1; aadA;*	(Pouget *et al.*, 2013)

									and/or *strA/B*	
			Dominican Republic, Vietnam, Thailand	2014	*K. pneumoniae* (E)	ND	ND	ND	-	(Zurfluh *et al.*, 2015)
			Spain	NA	*E. coli* (A)	C	-	-	CTX-M-9 or CMY-2	(Blanc *et al.*, 2006)
			Egypt	2011	*E. coli* (A)	C	No	-	*dfrA17-aadA5*; TEM-1; CTX-M-15; OXA-1; *tet(A), tet(E), qnrB2, aac(6)-Ib-cr; qnrA1, tet(E);* or *tet(B)*	(Ahmed *et al.*, 2013)
			Mexico	2001	*E. coli* (A)	ND	ND	ND	-	(Rocha-Gracia *et al.*, 2015)
			Netherlands	2010	*E. coli* (E)	ND	ND	ND	-	(Cohen Stuart *et al.*, 2012)
			Czech Republic	2005	*E. coli* (A)	ND	ND	ND	*dhfr17-aadA5;* or *dhfr1-aadA1*	(Dolejská *et al.*, 2009)
			Netherlands	2006	*E. coli* (A)	IncK	Yes	ND	TEM-1 or TEM-135-	(Dierikx *et al.*, 2010)
			Portugal	2003	*K. pneumoniae* (A)	P	Yes	ND	*dfrA12-orfF-aadA2*	(Machado *et al.*, 2008)
			Japan	2004-2006	*E. coli* (A)	P	Yes	ND	TEM-1	(Hiroi *et al.*, 2011)
***bla*$_{\mathbf{SHV-2a}}$**	X98102	7.6	Germany	1987-1988	*K. pneumoniae*	pZMP1	Yes	66	ND	(Podbielski *et al.*, 1991)
			Australia	1989-2015	*K. pneumoniae*	ND	ND	ND	ND	(Hall *et al.*, 2016)
			Croatia	1994-1995	*K. pneumoniae*	ND	ND	ND	ND	(Bedenic *et al.*, 2001)
			Korea	2004	*K. pneumoniae*	P	Yes	ND	DHA-1	(Song *et al.*, 2006)
			Slovenia	1998-2002	*K. pneumoniae; E. coli; E. cloacae; C. freundii*	C and P	Yes	120-150	TEM-15	(Zarnayová *et al.*, 2005)
			Greece	2006	*E. cloacae*	P	Yes	40	VIM-4; *aacA7; dhfrA1; aadA1; sul1*	(Ikonomidis *et al.*, 2007)
			Hungary	1998-2002	*K. pneumoniae; K. oxytoca*	IncR	Yes	Variable	-	(Damjanova *et al.*, 2007)
			Mexico	2006	*K. pneumoniae*	P	Yes	60-100	*aac(6')-Ib*	(Silva-Sanchez *et al.*, 2011)
			France	2011	*P. aeruginosa*	C (IS*26*)	-	-	VIM-4; *aacA*	(Jeannot *et al.*, 2013)
			DR Congo	2007-2011	*S. typhimurium; S. enterica* 4,5	ND	ND	ND	ND	(Lunguya *et al.*, 2013)
			Egypt	2002-2003	*K. pneumoniae*	P	ND	ND	TEM-1 or CTX-M-15	(Newire *et al.*, 2013)
			Tunisia	2005-2007	*P. aeruginosa*	C (IS*26*)	-	-		(Mansour *et al.*, 2009)
			Thailand	2014	*C. sakazakii; K. pneumoniae* (E)	ND	ND	ND	-	(Zurfluh *et al.*, 2015)
			Canada	2006-2007	*E. coli* (E)*; S. enterica* (A)	IncI1; IncP	-	-	*tetA/B; sul1; dfrA1; aadA;* and/or *strA/B*	(Pouget *et al.*, 2013)
			Japan	2004	*E. coli* (A)	C	-	-	-	(Hiki *et al.*, 2013)

bla$_{SHV-3}$	KX092356	7	France	1986	*K. pneumoniae*	pUD18	Yes	180	ND	(Nicolas *et al.*, 1989)
			USA	1993-1995	*E. aerogenes*	C and/or P	ND	10-50	AmpC	(Pitout *et al.*, 1998)
			USA	2008-2009	*E. coli* (A)	C	-	-	CTX-M-15; CTX-M-24; CMY-2; and/or TEM-1 (on various plasmid)	(Shaheen *et al.*, 2011)
bla$_{SHV-4}$	NA	7.8	France	1987	*K. pneumoniae*	P	Yes	180	ND	(Péduzzi *et al.*, 1989)
			France	1993	*E. aerogenes*	P (different profiles)	ND	ND	ND	(Arpin *et al.*, 1996)
			France	1988-1989	*C. diversus*	P (different profiles)	Yes	ND	ND	(El Harrif-Heraud *et al.*, 1997)
			USA	1993-1995	*E. aerogenes*	P	ND	Variable	AmpC	(Pitout *et al.*, 1998)
			Turkey	1997	*E. coli;*	ND	ND	ND	ND	(Durmaz *et al.*, 2001)
			Turkey	1994	*K. pneumoniae*	P	ND	Variable	SHV-3; SHV-5	(Yuan *et al.*, 1998)
			France	2002-2005	*E. cloacae*	P	ND	ND	TEM-4; variable ESBL-like	(Biendo *et al.*, 2008)
			Iraq	2008-2010	*E. coli*	ND	ND	ND	CTX-M or OXA	(Al-Mayahie, 2013)
			Belgium	1994	*K. pneumoniae*	P	ND	ND	ND	(Yuan *et al.*, 1998)
bla$_{SHV-5}$	X55640	8.2	Chile	1987	*K. pneumoniae*	pAFF1	No	150	ND	(Gutmann *et al.*, 1989; Billot-Klein *et al.*, 1990)
			China	2003-2004	*E. cloacae*	P	Yes	ND	ND	(Liu *et al.*, 2008)
			Taiwan	1997-2001	*K. pneumoniae*	C	-	-	TEM-1 or SHV-1	(Lin *et al.*, 2006)
			Canada	1999-2000	*K. pneumoniae*	P	Yes	Variable	ND	(Mulvey *et al.*, 2004)
			USA	1993-1995	*E. aerogenes*	C	-	-	AmpC	(Pitout *et al.*, 1998)
			Belgium	1994	*K. pneumoniae*	P	ND	ND	ND	(Yuan *et al.*, 1998)
			Greece	2012	*P. stuartii*	IncA/C	Yes	130	SHV-5; VIM-1; VEB-1; OXA-10; TEM-1	(Giakkoupi *et al.*, 2015)
			Mexico	2010-2011	*K. pneumoniae; E. coli*	P	ND	Variable	CTX-M-15	(Morfín-Otero *et al.*, 2013)
			Greece	2000	*S. enterica* Brandenburg	P	Y	120	-	(Politi *et al.*, 2005)
			China	2005-2011	*S. marcescens*	P	ND	ND	CTX-M-14; OXA-1; ACT-1; and/or DHA-1 *aac(6=)-Ib-cr*	(Yang *et al.*, 2012)
			USA	2004	*A. baumanii*	C (IS*26*)	-	-	-	(Naas *et al.*, 2007)
			Greece	1998-2002	*P. aeruginosa*	C	-	-	-	(Poirel *et al.*, 2004)
			Uruguay	2009	*S. marcescens*	IncP; IncFIB; IncFIC; IncA/C	Yes	ND	*aadA1/aac(6')Ib7*	(García-Fulgueiras *et al.*, 2011)
			Japan	2004	*E. coli* (A)	C	-	-	-	(Hiki *et al.*, 2013)
			Tunisia	2006-2007	*E. coli* (A)	IncI1, IncFIB, IncFII	ND	ND	ND	(Jouini *et al.*, 2013)

			Japan	2011	*E. coli* (A)	P	ND	ND	CMY-2	(Kameyama *et al.*, 2013)
			Spain	NA	*E. coli* (A)	C	-	-	CTX-M-9 or CMY-2	(Blanc *et al.*, 2006)
			Portugal	2008	*E. coli* (A)	ND	ND	ND	TEM-1b	(Pinto *et al.*, 2010)
			Australia	1991-1995	*K. pneumoniae*	ND	ND	ND	ND	(Schooneveldt *et al.*, 1998)
***bla*$_{\mathbf{SHV-6}}$**	Y11069.1	7.6	France	1991	*K. pneumoniae*	pSLH06	Yes	180	ND	(Arlet *et al.*, 1991)
***bla*$_{\mathbf{SHV-7}}$**	U20270	7.6	USA	1993	*E. coli*	P	Yes	10	ND	(Bradford *et al.*, 1995)
			China	NA	*E. coli; C. freundii*	C	-	-	TEM-1; KPC-3; CTX-M-14	(Li *et al.*, 2011)
			USA	1999-2002	*K. pneumoniae*	P	Yes	ND	*qnr*	(Wang *et al.*, 2004)
***bla*$_{\mathbf{SHV-8}}$**	U92041	7.6	USA	1990	*E. coli*	C	-	-	-	(Rasheed *et al.*, 1997)
			Australia	1991-1995	*K. pneumoniae*	ND	ND	ND	ND	(Schooneveldt *et al.*, 1998)
***bla*$_{\mathbf{SHV-9}}$**	S82452.1	8.2	Greece	1995	*E. coli; K. pneumoniae; S. marcescens*	pK318-1; pE77-1; pS24-1	Yes	ND	ND	(Prinarakis *et al.*, 1996)
			China	1998-2002	*K. pneumoniae*	P	ND	ND	ND	(Yu *et al.*, 2007)
			Australia	2000	*S. enterica* Typhimurium	ND	ND	ND	OXA-30; CMY-7	(Hanson *et al.*, 2002)
			Turkey	NA	*S. enterica* Paratyphi B	ND	ND	ND	ND	(Budak *et al.*, 2009)
***bla*$_{\mathbf{SHV-12}}$**	JX268741	8.2	Switzerland	1993-1995	*E. coli; K. pneumoniae*	P	Yes	80	ND	(Nüesch-Inderbinen *et al.*, 1997)
			Italy	2014	*A. caviae*	pAOUC-AA14	Yes	150	VIM-1; *dfrA14*	(Antonelli *et al.*, 2016)
			Venezuela	2011	*Salmonella* Give	P	Yes	ND	TEM-1; *qnr*	(Gonzalez and Araque, 2013)
			China	2003-2004	*E. cloacae*	P	Yes	ND	ND	(Liu *et al.*, 2008)
			Portugal	2006	*K. pneumoniae*	ND	ND	ND	TEM-1	(Mendonça *et al.*, 2009)
			Brazil	2005-2007	*K. pneumoniae*	ND	ND	ND	CTX-M-59; TEM-1	(Tollentino *et al.*, 2010)
			Portugal	2003-2004	*K. oxytoca*	P	ND	Variable	-	(Machado *et al.*, 2007)
			Canada	1999-2000	*K. pneumoniae*	P	Yes	Variable	ND	(Mulvey *et al.*, 2004)
			USA	1999-2004	*S. enterica* Senftenberg; *S. enterica* Typhimurium	ND	ND	ND	ND	(Whichard *et al.*, 2007)
			Saudi Arabia	2011	*K. pneumoniae*	ND	ND	ND	ND	(Hassan and Abdalhamid, 2014)
			Malawi	2004-2005	*K. oxytoca; E. cloacae; K. pneumoniae*	ND	ND	ND	ND	(Gray *et al.*, 2006)
			Thailand	2004-2005	*E. coli; K. pneumoniae*	P	Yes	ND	CTX-M-14; TEM-1; SHV-12; OXA-10; VEB-1 or CTX-M-15	(Kiratisin *et al.*, 2008)
			Mali	2004-2006	*E. coli; K. pneumoniae; E. cloacae*	ND	ND	ND	CTX-M-15	(Duval *et al.*, 2009)

			France	2002-2005	*E. cloacae*	P	ND	ND	TEM-24; CTX-M-9 or variable ESBL-like	(Biendo *et al.*, 2008)
			Korea	1997	*K. pneumoniae*	pK7746-C1 (IS*26*)	Yes	123	ND	(Kim *et al.*, 2002)
			Spain	NA	*S. enterica* Bredeney	pB1004 (IncHI)	Yes	315	TEM-1; *qnrB2*	(Gutierrez *et al.*, 2009)
			Korea	2008	*P. mirabilis*	C	-	-	CTX-M-14; DHA-1	(Song *et al.*, 2011)
			Italy	2005	*Klebsiella oxytoca*	IncN	Yes	55	VIM-1; SHV-12, *qnrS1; aacA4; dfrA14*	(Carattoli *et al.*, 2010)
			Japan	NA	*P. aeruginosa*	C (IS*26*)	-	-	*aac(6')-Ib*	(Uemura *et al.*, 2010)
			Senegal	2000	*E. coli; S. enterica* Agona and Kentucky	IncHI2, IncF	Yes	Variable	*qnrB2, Aac(6')-Ib-cr*	(Harrois *et al.*, 2014)
			Dominican Republic, Thailand	2014	*C. sakazakii; E. coli; K. pneumoniae*	ND	ND	ND	-	(Zurfluh *et al.*, 2015)
			Portugal	2004-2005	*C. freundii* (A)	P	Yes	ND	-	(Machado *et al.*, 2008)
			Japan	2004	*E. coli* (A)	C	-	-	-	(Hiki *et al.*, 2013)
			Croatia	2009-2010	*Aeromonas spp* (E)	C	-	-	CTX-M-15	(Maravić *et al.*, 2013)
			Portugal	NA	*E. coli* (E)	IncI1 (IS*26*; IS*903*)	ND	ND	-	(Jones-Dias *et al.*, 2015)
			Japan	2002-2003	*S. enterica* Manhattan (E)	P	ND	Variable	ND	(Noda *et al.*, 2015)
			UK	2010	*K. pneumoniae* (E)	IncK/B	No	ND	TEM-1	(Timofte *et al.*, 2014)
			Germany	2011-2012	*E. coli* (A)	C	-	-	CMY-2; TEM-1; OXA-48; *aac(6')-Ib-cr, qnrB2*	(Stolle *et al.*, 2013)
			Netherlands	2009	*E. coli* (A)	IncFIB, IncK, IncB/O, IncColE	Yes	ND	*aadA2*	(Dierikx *et al.*, 2013)
			Egypt	2006-2007	*S. enterica* Typhimurium (A)	ND	ND	ND	TEM-1; *qnrB*	(Ahmed *et al.*, 2009)
			Switzerland	2012	*Enterobacteriaceae* (E)	ND	ND	ND	CTX-M	(Zurfluh *et al.*, 2013)
			Croatia	2009-2013	*K. pneumoniae; E. coli; E. cloacae; E. aerogenes; K. oxytoca; R. terrigena* (E)	P	ND	ND	TEM-1 and/or CTX-M-15	(Maravic *et al.*, 2015)
			Spain	2013-2014	*E. coli* (A)	P	ND	ND	TEM-1	(Alcala *et al.*, 2015)
bla$_{SHV-13}$	AF164577	7.6	Netherlands	1994	*K. pneumoniae*	P	Yes	170	ND	(Yuan *et al.*, 2000)
bla$_{SHV-15}$	AJ011428.2	ND	India	1998	*E. coli*	ND	ND	ND	ND	http://www.lahey.org/studies/
bla$_{SHV-16}$	AF072684.2	7.6	France	1996	*K. pneumoniae*	P	Yes	>100	-	(Arpin *et al.*, 2001)
bla$_{SHV-18}$	AF132290	7.8	USA	1994	*K. pneumoniae*	P	Yes	80	ND	(Rasheed *et al.*, 2000)
bla$_{SHV-23}$	AF117747	ND	South Africa	1990	*K. pneumoniae*	ND	ND	ND	ND	(Essack *et al.*, 2004)
bla$_{SHV-24}$	AB023477	7.5	Japan	1996	*E. coli*	pCAZR001	Yes	150	ND	(Kurokawa *et al.*, 2000)

bla$_{SHV-27}$	AF293345.1	8.2	Brazil	1999	*K. pneumoniae*	C	-	-	ND	(Corkill *et al.*, 2001)
			Portugal	1999	*K. pneumoniae*	ND	ND	ND	-	(Mendonça *et al.*, 2009)
			Taiwan	1997-2001	*K. pneumoniae*	C	-	-	TEM-116	(Lin *et al.*, 2006)
			Malawi	2004-2005	*K. pneumoniae*	P	Yes	ND	ND	(Gray *et al.*, 2006)
			Japan	2000-2002	*K. pneumoniae*	P	ND	ND	DHA-1; TEM-1; CMY-2 or IMP	(Muratani *et al.*, 2006)
			Thailand	2004-2005	*K. pneumoniae*	P	Yes	ND	TEM-1; CTX-M-14 or CTX-M-15	(Kiratisin *et al.*, 2008)
			Tunisia	2006	*K. pneumoniae*	P (IS*Ecp*1)	Yes	70	CTX-M-15; TEM-1b; OXA-1, *dfr*A5; *ere*A2	(Abbassi *et al.*, 2008)
			Mali	2004-2006	*E. coli*	ND	ND	ND	CTX-M-15	(Duval *et al.*, 2009)
			Tunisia	2009	*E. cloacae*	ND	ND	ND	CTX-M-15, SHV-12	(Hammami *et al.*, 2011)
			China	2007-2009	*K. pneumoniae* (A)	ND	ND	ND	ND	(Zou *et al.*, 2011)
			China	2010	*E. coli* (A)	ND	ND	ND	ND	(Jiang *et al.*, 2012)
			Japan	2007-2009	*K. pneumoniae* (E)	ND	ND	ND	ND	(Hammad and Shimamoto, 2011)
bla$_{SHV-30}$	AY661885	6.7	USA	2003	*E. cloacae*	P	ND	9.4	AmpC, TEM-1, SHV-7	(Szabó *et al.*, 2005b)
			Canada	1999-2000	*K. pneumoniae*	P	Yes	Variable	ND	(Mulvey *et al.*, 2004)
			USA	2002	*S. enterica* Mbandaka	ND	ND	ND	ND	(Whichard *et al.*, 2007)
bla$_{SHV-31}$	AY277255	7.8	Netherlands	2001	*K. pneumoniae*	C	-	-	-	(Mazzariol *et al.*, 2007)
			Taiwan	ND	*K. pneumoniae*	P	Yes	ND	CMY-2; DHA-1	(Tang *et al.*, 2015)
			Brazil	2005-2007	*K. pneumoniae*	ND	ND	ND	-	(Tollentino *et al.*, 2010)
			Iran	2006-2007	*K. pneumoniae*	ND	ND	ND	ND	(Feizabadi *et al.*, 2010)
bla$_{SHV-34}$	AY036620	ND	USA	1998-2000	*C. koseri; E. coli; K. pneumoniae*	pOZ185	Yes	>100	ND	(Heritage *et al.*, 2003)
bla$_{SHV-38}$	AY079099	7.6	France	2001	*K. pneumoniae*	C	-	-	-	(Poirel *et al.*, 2003)
			Portugal	1999	*K. pneumoniae*	C	ND	ND	SHV-1; TEM-24	(Mendonça *et al.*, 2009)
			Brazil	2005-2007	*K. pneumoniae*	ND	ND	ND	CTX-M-2; TEM-1	(Tollentino *et al.*, 2010)
bla$_{SHV-40}$	AF535128	7.6	Canada	1999-2000	*K. pneumoniae*	ND	ND	ND	ND	(Mulvey *et al.*, 2004)
			Brazil	2004	*K. pneumoniae*	ND	ND	ND	TEM-116;<br>GES-7	(Dropa *et al.*, 2010)
bla$_{SHV-41}$	AF535129	7.6	Canada	1999-2000	*K. pneumoniae*	ND	ND	ND	ND	(Mulvey *et al.*, 2004)
			Taiwan	1997-2001	*K. pneumoniae*	C	-	-	TEM-116	(Lin *et al.*, 2006)
bla$_{SHV-42}$	AF535130	7.6	Canada	1999-2000	*K. pneumoniae*	ND	ND	ND	ND	(Mulvey *et al.*, 2004)

			India	2001-2002	*K. pneumoniae*	P	ND	ND	ND	(Dhara and Tripathi, 2014)
bla$_{\text{SHV-45}}$	AF547625	8.2	Brazil	NA	*K. pneumoniae*	IncA/C	ND	97-145	CTX-M-2; SHV-27	(Dropa, 2015)
bla$_{\text{SHV-46}}$	AY210887	8.2	New York	1998	*K. oxytoca*	P	Yes	70	TEM-1; OXY-2; KPC-2; OXA (?)	(Yigit *et al.*, 2003)
bla$_{\text{SHV-55}}$	DQ054528	ND	Portugal	NA	*K. pneumoniae*	ND	No	-	TEM1	(Mendonça *et al.*, 2006)
			Portugal	2003	*K. pneumoniae*	P	No	-	-	(Machado *et al.*, 2007)
			Brazil	NA	*K. pneumoniae*	IncA/C	ND	63.5/112/209	CTX-M-2; SHV-28	(Dropa, 2015)
bla$_{\text{SHV-57}}$	AY223863	8.3	Taiwan	1998	*E. coli*	pMTY512	Yes	40-60	ND	(Ma *et al.*, 2005)
			China	2006-2007	*E. coli*	ND	ND	ND	ND	(Tian *et al.*, 2012)
bla$_{\text{SHV-64}}$	DQ174304	ND	China	2000-2002	*K. pneumoniae*	ND	ND	ND	ND	(Zuo *et al.*, 2006)
bla$_{\text{SHV-66}}$	DQ174306	ND	China	2000-2002	*K. pneumoniae*	ND	ND	ND	ND	(Zuo *et al.*, 2006)
bla$_{\text{SHV-70}}$	DQ013287	7.6	China	2003-2004	*E. cloacae*	pEC04	Yes	ND	ND	(Ling *et al.*, 2006)
			China	2003-2004	*E. cloacae*	P	Yes	ND	ND	(Liu *et al.*, 2008)
bla$_{\text{SHV-86}}$	DQ328802	8.2	Colombia	2003	*K. pneumoniae*	P	Yes	ND	ND	(Espinal *et al.*, 2010)
bla$_{\text{SHV-90}}$	NA	8.2	Portugal	2003	*K. pneumoniae*	ND	ND	ND	ND	(Machado *et al.*, 2007)
bla$_{\text{SHV-91}}$	NA	7.6	Portugal	2003	*K. pneumoniae*	ND	ND	ND	ND	(Machado *et al.*, 2007)
bla$_{\text{SHV-98}}$	AM941844	7.6	Algeria	2005	*K. pneumoniae*	ND	ND	ND	ND	(Ramdani-Bouguessa *et al.*, 2011)
bla$_{\text{SHV-99}}$	AM941845	7.8	Algeria	2005	*K. pneumoniae*	ND	ND	ND	ND	(Ramdani-Bouguessa *et al.*, 2011)
bla$_{\text{SHV-100}}$	AM941846	7.2	Algeria	2005	*K. pneumoniae*	ND	ND	ND	ND	(Ramdani-Bouguessa *et al.*, 2011)
bla$_{\text{SHV-102}}$	EU024485	ND	Spain	2003-2004	*E. coli*	ND	ND	ND	ND	(Vinue *et al.*, 2008)
bla$_{\text{SHV-104}}$	EU274581	7,3/8,6	Tunisia	2004	*K. pneumoniae*	pML2011	Yes	50	ND	(Ben Achour *et al.*, 2014)
bla$_{\text{SHV-105}}$	FJ194944	ND	USA	NA	*K. pneumoniae*	ND	ND	ND	SHV-1; SHV-5	(Jones *et al.*, 2009)
bla$_{\text{SHV-106}}$	AM941847	7.6	Portugal	1999	*K. pneumoniae*	ND	ND	ND	TEM-1; CTX-M-32	(Mendonça *et al.*, 2009)
			Portugal	2006	*K. pneumoniae*	ND	ND	ND	CTX-M-32 or TEM-1	(Mendonça *et al.*, 2009)
bla$_{\text{SHV-128}}$	GU932590	8.6	Tunisia	2009	*E. cloacae*	IncFII (IS*26*)	Yes	100	ND	(Bourouis *et al.*, 2015)
bla$_{\text{SHV-129}}$	GU827715	ND	Italy	2008	*E. coli*	pEc6-66	ND	ND	ND	(Lascols *et al.*, 2012)
bla$_{\text{SHV-134}}$	HM559945	ND	Spain	2009	*K. pneumoniae*	IncFIIA (IS*26*)	Yes	75	VIM-1; *aac*(6')-*Ib; dhfrII; aadA1; catB2; TEM-1; aac(3')-Iia*	(Sanchez-Romero *et al.*, 2012)
bla$_{\text{SHV-183}}$	HG934764	ND	NA	NA	*E. cloacae*	ND	ND	ND	ND	http://www.lahey.org/studies/

*Isolation or first description; [§]A: animal sample; E: environmental sample
[¥]P: plasmid; C: Chromosome; when known plasmid name or Inc group are provided and Insertion Sequences indicated;
[#]For some enzymes (SHV2, SHV2a, SHV-3, SHV4, SHV-5, SHV-12) only representative references are reported;
NA: not available; ND: not determined.

References

Abbassi, M.S., Torres, C., Achour, W., Vinué, L., Sáenz, Y., Costa, D., et al. (2008). Genetic characterisation of CTX-M-15-producing *Klebsiella pneumoniae* and *Escherichia coli* strains isolated from stem cell transplant patients in Tunisia. *Int. J. Antimicrob. Agents* 32**,** 308-314.

Ahmed, A.M., Shimamoto, T., and Shimamoto, T. (2013). Molecular characterization of multidrug-resistant avian pathogenic *Escherichia coli* isolated from septicemic broilers. *Int. J. Med. Microbiol.* 303**,** 475-483.

Ahmed, A.M., Younis, E.E.A., Ishida, Y., and Shimamoto, T. (2009). Genetic basis of multidrug resistance in *Salmonella* enterica serovars Enteritidis and Typhimurium isolated from diarrheic calves in Egypt. *Acta Trop.* 111**,** 144-149.

Al-Mayahie, S.M. (2013). Phenotypic and genotypic comparison of ESBL production by vaginal *Escherichia coli* isolates from pregnant and non-pregnant women. *Ann. Clin. Microbiol. Antimicrob.* 12**,** 1-7.

Alcala, L., Alonso, C.A., Simon, C., Gonzalez-Esteban, C., Oros, J., Rezusta, A., et al. (2015). Wild Birds, Frequent Carriers of Extended-Spectrum beta-Lactamase (ESBL) Producing Escherichia coli of CTX-M and SHV-12 Types. *Microb Ecol*.

Andres, P., Petroni, A., Faccone, D., Pasterán, F., Melano, R., Rapoport, M., et al. (2005). Extended-spectrum β-lactamases in *Shigella flexneri* from Argentina: first report of TOHO-1 outside Japan. *Int. J. Antimicrob. Agents* 25**,** 501-507.

Antonelli, A., D'andrea, M.M., Montagnani, C., Bartolesi, A.M., Di Pilato, V., Fiorini, P., et al. (2016). Newborn bacteraemia caused by an *Aeromonas caviae* producing the VIM-1 and SHV-12 β-lactamases, encoded by a transferable plasmid. *J. Antimicrob. Chemother.* 71**,** 272-274.

Arlet, G., Rouveau, M., Bengoufa, D., Nicolas, M.H., and Philippon, A. (1991). Novel transferable extended-spectrum β-lactamase (SHV-6) from *Klebsiella pneumoniae* conferring selective resistance to ceftazidime. *FEMS Microbiol. Lett.* 81**,** 57-62.

Arpin, C., Coze, C., Rogues, A.M., Gachie, J.P., Bebear, C., and Quentin, C. (1996). Epidemiological study of an outbreak due to multidrug-resistant *Enterobacter aerogenes* in a medical intensive care unit. *J. Clin. Microbiol.* 34**,** 2163-2169.

Arpin, C., Labia, R., Andre, C., Frigo, C., El Harrif, Z., and Quentin, C. (2001). SHV-16, a β-lactamase with a pentapeptide duplication in the omega loop. *Antimicrob. Agents Chemother.* 45**,** 2480-2485.

Bedenic, B., Randegger, C.C., Stobberingh, E., and Hachler, H. (2001). Molecular epidemiology of extended-spectrum beta-lactamases from *Klebsiella pneumoniae* strains isolated in Zagreb, Croatia. *Eur. J. Clin. Microbiol. Infect. Dis.* 20**,** 505-508.

Ben Achour, N., Belhadj, O., Galleni, M., Ben Moussa, M., and Mercuri, P.S. (2014). Study of a natural mutant SHV-type β-lactamase, SHV-104, from *Klebsiella pneumoniae*. *Int. J. Microbiol.* 2014**,** 6.

Biendo, M., Manoliu, C., Laurans, G., Castelain, S., Canarelli, B., Thomas, D., et al. (2008). Molecular typing and characterization of extended-spectrum TEM, SHV and CTX-M β-lactamases in clinical isolates of *Enterobacter cloacae*. *Res. Microbiol.* 159**,** 590-594.

Billard-Pomares, T., Fouteau, S., Jacquet, M.E., Roche, D., Barbe, V., Castellanos, M., et al. (2014). Characterization of a P1-like bacteriophage carrying an SHV-2 extended-spectrum β-lactamase from an *Escherichia coli* strain. *Antimicrob. Agents Chemother.* 58**,** 6550-6557.

Billot-Klein, D., Gutmann, L., and Collatz, E. (1990). Nucleotide sequence of the SHV-5 beta-lactamase gene of a *Klebsiella pneumoniae* plasmid. *Antimicrob. Agents Chemother.* 34**,** 2439-2441.

Blanc, V., Mesa, R., Saco, M., Lavilla, S., Prats, G., Miró, E., et al. (2006). ESBL- and plasmidic class C β-lactamase-producing *E. coli* strains isolated from poultry, pig and rabbit farms. *Vet. Microbiol.* 118**,** 299-304.

Bourouis, A., Ben Moussa, M., and Belhadj, O. (2015). Multidrug-resistant phenotype and isolation of a novel SHV- beta-lactamase variant in a clinical isolate of *Enterobacter cloacae*. *J. Biomed. Sci.* 22**,** 1-7.

Bradford, P.A., Urban, C., Jaiswal, A., Mariano, N., Rasmussen, B.A., Projan, S.J., et al. (1995). SHV-7, a novel cefotaxime-hydrolyzing beta-lactamase, identified in *Escherichia coli* isolates from hospitalized nursing home patients. *Antimicrob. Agents Chemother.* 39**,** 899-905.

Budak, F., Nordmann, P., Girlich, D., and Gür, D. (2009). Characterization of extended-spectrum beta-lactamase-producing *Salmonella* isolates in a children's hospital in Ankara--first report of SHV-2a and SHV-9 in *Salmonella* spp. from Turkey. *Turk. J. Pediatr.* 51**,** 28-34.

Cao, V., Lambert, T., Nhu, D.Q., Loan, H.K., Hoang, N.K., Arlet, G., et al. (2002). Distribution of extended-spectrum β-lactamases in clinical isolates of *Enterobacteriaceae* in Vietnam. *Antimicrob. Agents Chemother.* 46**,** 3739-3743.

Carattoli, A., Aschbacher, R., March, A., Larcher, C., Livermore, D.M., and Woodford, N. (2010). Complete nucleotide sequence of the IncN plasmid pKOX105 encoding VIM-1, QnrS1 and SHV-12 proteins in *Enterobacteriaceae* from Bolzano, Italy compared with IncN plasmids encoding KPC enzymes in the USA. *J. Antimicrob. Chemother.* 65**,** 2070-2075.

Chen, Y.-T., Shu, H.-Y., Li, L.-H., Liao, T.-L., Wu, K.-M., Shiau, Y.-R., et al. (2006). Complete nucleotide sequence of pK245, a 98-kilobase plasmid conferring quinolone resistance and extended-spectrum-β-lactamase activity in a clinical *Klebsiella pneumoniae* isolate. *Antimicrob. Agents Chemother.* 50**,** 3861-3866.

Cohen Stuart, J., Van Den Munckhof, T., Voets, G., Scharringa, J., Fluit, A., and Hall, M.L.-V. (2012). Comparison of ESBL contamination in organic and conventional retail chicken meat. *Int. J. Food Microbiol.* 154**,** 212-214.

Corkill, J.E., Cuevas, L.E., Gurgel, R.Q., Greensill, J., and Hart, C.A. (2001). SHV-27, a novel cefotaxime-hydrolysing β-lactamase, identified in *Klebsiella pneumoniae* isolates from a Brazilian hospital. *J. Antimicrob. Chemother.* 47**,** 463-465.

Damjanova, I., Tóth, Á., Pászti, J., Jakab, M., Milch, H., Bauernfeind, A., et al. (2007). Epidemiology of SHV-type β-lactamase-producing *Klebsiella* spp. from outbreaks in five geographically distant Hungarian neonatal intensive care units: widespread dissemination of epidemic R-plasmids. *Int. J. Antimicrob. Agents* 29**,** 665-671.

Decré, D., Burghoffer, B., Gautier, V., Petit, J.-C., and Arlet, G. (2004). Outbreak of multi-resistant *Klebsiella oxytoca* involving strains with extended-spectrum β-lactamases and strains with extended-spectrum activity of the chromosomal β-lactamase. *J. Antimicrob. Chemother.* 54**,** 881-888.

Dhara, L., and Tripathi, A. (2014). Genetic and structural insights into plasmid-mediated extended-spectrum β-lactamase activity of CTX-M and SHV variants among pathogenic *Enterobacteriaceae* infecting Indian patients. *Int. J. Antimicrob. Agents* 43**,** 518-526.

Dierikx, C., Van Der Goot, J., Fabri, T., Van Essen-Zandbergen, A., Smith, H., and Mevius, D. (2013). Extended-spectrum-β-lactamase- and AmpC-β-lactamase-producing *Escherichia coli* in Dutch broilers and broiler farmers. *J. Antimicrob. Chemother.* 68**,** 60-67.

Dierikx, C., Van Essen-Zandbergen, A., Veldman, K., Smith, H., and Mevius, D. (2010). Increased detection of extended spectrum beta-lactamase producing *Salmonella enterica* and *Escherichia coli* isolates from poultry. *Vet. Microbiol.* 145**,** 273-278.

Dolejská, M., Bierošová, B., Kohoutová, L., Literák, I., and Čížek, A. (2009). Antibiotic-resistant *Salmonella* and *Escherichia coli* isolates with integrons and extended-spectrum beta-lactamases in surface water and sympatric black-headed gulls. *J. Appl. Microbiol.* 106**,** 1941-1950.

Dropa, M., Balsalobre, L.C., Lincopan, N., Mamizuka, E.M., Cassettari, V.C., Matté, G.R., et al. (2010). Emergence of *Klebsiella pneumoniae* carrying the novel extended-spectrum β-lactamase gene variants *bla*SHV-40, *bla*TEM-116 and the class 1 integron associated *bla*GES-7 in Brazil. *Clin. Microbiol. Infect.* 16**,** 630-632.

Dropa, M., Balsalobre, L., Lincopan, N., Matte', G., Matte'm. (2015). Complex class 1 integrons harboring CTX-M-2-encoding genes in clinical *Enterobacteriaceae* from a hospital in Brazil. *J. Infect. Dev. Ctries* 9**,** 890-897.

Durmaz, R., Durmaz, B., Koroglu, M., and Tekerekoglu, M.S. (2001). Detection and typing of extended-spectrum β-lactamases in clinical isolates of the family *Enterobacteriaceae* in a medical center in Turkey. *Microb. Drug Res.* 7**,** 171-175.

Duval, V., Maiga, I., Maiga, A., Guillard, T., Brasme, L., Forte, D., et al. (2009). High prevalence of CTX-M-type β-lactamases among clinical isolates of *Enterobacteriaceae* in Bamako, Mali. *Antimicrob. Agents Chemother.* 53**,** 4957-4958.

El Harrif-Heraud, Z., Arpin, C., Benliman, S., and Quentin, C. (1997). Molecular epidemiology of a nosocomial outbreak due to SHV-4-producing strains of *Citrobacter diversus*. *J. Clin. Microbiol.* 35**,** 2561-2567.

Espinal, P., Garza-Ramos, U., Reyna, F., Rojas-Moreno, T., Sanchez-Perez, A., Carrillo, B., et al. (2010). Identification of SHV-type and CTX-M-12 extended-spectrum beta-lactamases (ESBLs) in multiresistant *Enterobacteriaceae* from Colombian Caribbean hospitals. *J. Chemother.* 22**,** 160-164.

Essack, S.Y., Hall, L.M.C., and Livermore, D.M. (2004). *Klebsiella pneumoniae* isolate from South Africa with multiple TEM, SHV and AmpC β-lactamases. *Int. J. Antimicrob. Agents* 23**,** 398-400.

Feizabadi, M.M., Mahamadi-Yeganeh, S., Mirsalehian, A., Mirafshar, S.-M., Mahboobi, M., Nili, F., et al. (2010). Genetic characterization of ESBL producing strains of *Klebsiella pneumoniae* from Tehran hospitals. *J. Infect. Dev. Ctries* 4.

García-Fulgueiras, V., Bado, I., Mota, M.I., Robino, L., Cordeiro, N.F., Varela, A., et al. (2011). Extended-spectrum β-lactamases and plasmid-mediated quinolone resistance in enterobacterial clinical isolates in the paediatric hospital of Uruguay. *J. Antimicrob. Chemother.* 66**,** 1725-1729.

Garza-Ramos, U., Martinez-Romero, E., and Silva-Sanchez, J. (2007). SHV-type extended-spectrum beta-lactamase (ESBL) are encoded in related plasmids from enterobacteria clinical isolates from Mexico. *Salud Publica Mex.* 49**,** 415-421.

Giakkoupi, P., Tryfinopoulou, K., Polemis, M., Pappa, O., Miriagou, V., and Vatopoulos, A. (2015). Circulation of a multiresistant, conjugative, IncA/C plasmid within the nosocomial *Providencia stuartii* population in the Athens area. *Diagn. Microbiol. Infect. Dis.* 82**,** 62-64.

González-Sanz, R., Herrera-León, S., De La Fuente, M., Arroyo, M., and Echeita, M.A. (2009). Emergence of extended-spectrum β-lactamases and AmpC-type β-lactamases in human *Salmonella* isolated in Spain from 2001 to 2005. *J. Antimicrob. Chemother.* 64**,** 1181-1186.

Gonzalez, F., and Araque, M. (2013). Association of transferable quinolone resistance determinant *qnr*B19 with extended-spectrum β-lactamases in *Salmonella* Give and *Salmonella* Heidelberg in Venezuela. *Int. J. Microbiol.* 2013**,** 6.

Gray, K.J., Wilson, L.K., Phiri, A., Corkill, J.E., French, N., and Hart, C.A. (2006). Identification and characterization of ceftriaxone resistance and extended-spectrum β-lactamases in Malawian bacteraemic *Enterobacteriaceae*. *J. Antimicrob. Chemother.* 57**,** 661-665.

Gutierrez, B., Herrera-Leon, S., Escudero, J.A., Hidalgo, L., Gonzalez-Sanz, R., Arroyo, M., et al. (2009). Novel genetic environment of qnrB2 associated with TEM-1 and SHV-12 on pB1004, an IncHI2 plasmid, in *Salmonella* Bredeney BB1047 from Spain. *J. Antimicrob. Chemother.* 64**,** 1334-1336.

Gutmann, L., Ferré, B., Goldstein, F.W., Rizk, N., Pinto-Schuster, E., Acar, J.F., et al. (1989). SHV-5, a novel SHV-type beta-lactamase that hydrolyzes broad-spectrum cephalosporins and monobactams. *Antimicrob. Agents Chemother.* 33**,** 951-956.

Hall, J.M., Ingram, P.R., Apos, Reilly, L.C., and Inglis, T.J.J. (2016). Temporal flux in β-lactam resistance among Klebsiella pneumoniae in Western Australia. *J. Med. Microbiol.* 65**,** 429-437.

Hammad, A.M., and Shimamoto, T. (2011). Asymptomatic intramammary infection with multidrug-resistant Gram-negative bacteria in a research dairy farm: incidence and genetic basis of resistance. *J. Vet. Med. Sci.* 73**,** 1089-1092.
Hammami, S., Boubaker, I.B.-B., Saidani, M., Lakhal, E., Hassen, A.B., Kamoun, A., et al. (2011). Characterization and molecular epidemiology of extended spectrum beta-lactamase producing *Enterobacter cloacae* isolated from a Tunisian hospital. *Microb. Drug Res.* 18**,** 59-65.
Hanson, N.D., Moland, E.S., Hossain, A., Neville, S.A., Gosbell, I.B., and Thomson, K.S. (2002). Unusual *Salmonella* enterica serotype *Typhimurium* isolate producing CMY-7, SHV-9 and OXA-30 β-lactamases. *J. Antimicrob. Chemother.* 49**,** 1011-1014.
Harrois, D., Breurec, S., Seck, A., Delauné, A., Hello, S.L., Gándara, M.P.D.L., et al. (2014). Prevalence and characterization of extended-spectrum β-lactamase-producing clinical *Salmonella enterica* isolates in Dakar, Senegal, from 1999 to 2009. *Clin. Microbiol. Infect.* 20**,** O109-O116.
Hassan, H., and Abdalhamid, B. (2014). Molecular characterization of extended-spectrum beta-lactamase producing *Enterobacteriaceae* in a Saudi Arabian tertiary hospital. *J. Infect. Dev. Ctries* 8**,** 282-288.
Heritage, J., Chambers, P.A., Tyndall, C., and Buescher, E.S. (2003). SHV-34: an extended-spectrum β-lactamase encoded by an epidemic plasmid. *J. Antimicrob. Chemother.* 52**,** 1015-1017.
Hiki, M., Usui, M., Kojima, A., Ozawa, M., Ishii, Y., and Asai, T. (2013). Diversity of plasmid replicons encoding the *bla*CMY-2 gene in broad-spectrum cephalosporin-resistant *Escherichia coli* from livestock animals in Japan. *Foodborne Pathog. Dis.* 10**,** 243-249.
Hiroi, M., Harada, T., Kawamori, F., Takahashi, N., Kanda, T., Sugiyama, K., et al. (2011). A survey of beta-lactamase-producing *Escherichia coli* in farm animals and raw retail meat in Shizuoka Prefecture, Japan. *Jpn. J. Infect. Dis.* 64**,** 153-155.
Ikonomidis, A., Spanakis, N., Poulou, A., Pournaras, S., Markou, F., and Tsakris, A. (2007). Emergence of carbapenem-resistant *Enterobacter cloacae* carrying VIM-4 metallo-β-lactamase and SHV-2a extended-spectrum β-lactamase in a conjugative plasmid. *Microb. Drug Res.* 13**,** 221-226.
Jeannot, K., Fournier, D., Müller, E., Cholley, P., and Plésiat, P. (2013). Clonal dissemination of *Pseudomonas aeruginosa* isolates producing extended-spectrum β-lactamase SHV-2a. *J. Clin. Microbiol.* 51**,** 673-675.
Jiang, H.-X., Tang, D., Liu, Y.-H., Zhang, X.-H., Zeng, Z.-L., Xu, L., et al. (2012). Prevalence and characteristics of β-lactamase and plasmid-mediated quinolone resistance genes in *Escherichia coli* isolated from farmed fish in China. *J. Antimicrob. Chemother.* 67**,** 2350-2353.
Jones-Dias, D., Manageiro, V., Martins, A.P., Ferreira, E., and Caniça, M. (2015). New class 2 integron In2-4 among IncI1-positive *Escherichia coli* isolates carrying ESBL and PMAβ genes from food animals in Portugal. *Foodborne Pathog. Dis.* 13**,** 36-39.
Jones, C.H., Ruzin, A., Tuckman, M., Visalli, M.A., Petersen, P.J., and Bradford, P.A. (2009). Pyrosequencing using the single-Nucleotide polymorphism protocol for rapid determination of TEM- and SHV-type extended-spectrum β-lactamases in clinical isolates and identification of the novel β-lactamase genes blaSHV-48, blaSHV-105, and blaTEM-155. *Antimicrob. Agents Chemother.* 53**,** 977-986.
Jouini, A., Slama, K.B., Klibi, N., Sallem, R.B., Estepa, V., Vinué, L., et al. (2013). Lineages and virulence gene content among extended-spectrum b-lactamase-producing *Escherichia coli* strains of food origin in Tunisia. *J. Food Prot.* 76**,** 323-327.
Kameyama, M., Chuma, T., Yabata, J., Tominaga, K., Iwata, H., and Okamoto, K. (2013). Prevalence and epidemiological relationship of CMY-2 AmpC beta-cactamase and CTX-M extended-spectrum beta-lactamase-producing *Escherichia coli* isolates from broiler farms in Japan. *J. Vet. Med. Sci.* 75**,** 1009-1015.
Kim, J., Shin, H.-S., Seol, S.-Y., and Cho, D.-T. (2002). Relationship between blaSHV-12 and blaSHV-2a in Korea. *J. Antimicrob. Chemother.* 49**,** 261-267.
Kiratisin, P., Apisarnthanarak, A., Laesripa, C., and Saifon, P. (2008). Molecular characterization and epidemiology of extended-spectrum- β-lactamase-producing *Escherichia coli* and *Klebsiella pneumoniae* isolates causing health care-associated infection in Thailand, where the CTX-M family is endemic. *Antimicrob. Agents Chemother.* 52**,** 2818-2824.
Kliebe, C., Nies, B.A., Meyer, J.F., Tolxdorff-Neutzling, R.M., and Wiedemann, B. (1985). Evolution of plasmid-coded resistance to broad-spectrum cephalosporins. *Antimicrob. Agents Chemother.* 28**,** 302-307.
Kurokawa, H., Yagi, T., Shibata, N., Shibayama, K., Kamachi, K., and Arakawa, Y. (2000). A new SHV-derived extended-spectrum β-Lactamase (SHV-24) that hydrolyzes ceftazidime through a single-amino-acid substitution (D179G) in the Ω-Loop. *Antimicrob. Agents Chemother.* 44**,** 1725-1727.
Lascols, C., Hackel, M., Hujer, A.M., Marshall, S.H., Bouchillon, S.K., Hoban, D.J., et al. (2012). Using nucleic acid microarrays to perform molecular epidemiology and detect novel β-lactamases: a snapshot of extended-spectrum β-lactamases throughout the world. *J. Clin. Microbiol.* 50**,** 1632-1639.
Li, G., Wei, Q., Wang, Y., Du, X., Zhao, Y., and Jiang, X. (2011). Novel genetic environment of the plasmid-mediated KPC-3 gene detected in *Escherichia coli* and *Citrobacter freundii* isolates from China. *Eur. J. Clin. Microbiol. Infect. Dis.* 30**,** 575-580.
Lin, T.-L., Tang, S.-I., Fang, C.-T., Hsueh, P.-R., Chang, S.-C., and Wang, J.-T. (2006). Extended-spectrum β-lactamase genes of *Klebsiella pneumoniae* strains in Taiwan: eecharacterization of *shv*-27, *shv*-41, and *tem*-116. *Microb. Drug Res.* 12**,** 12-15.
Ling, B.-D., Liu, G., Xie, Y.-E., Zhou, Q.-X., Zhao, T.-K., Li, C.-Q., et al. (2006). Characterisation of a novel extended-spectrum β-lactamase, SHV-70, from a clinical isolate of *Enterobacter cloacae* in China. *Int. J. Antimicrob. Agents* 27**,** 355-356.
Liu, G., Ling, B., Zeng, Y., Lin, L., Xie, Y., and J, L. (2008). Molecular characterization of extended-spectrum beta-lactamases produced by clinical isolates of *Enterobacter cloacae* from a teaching hospital in China. *Jpn. J. Infect. Dis.* 61**,** 286-289.
Lunguya, O., Lejon, V., Phoba, M.-F., Bertrand, S., Vanhoof, R., Glupczynski, Y., et al. (2013). Antimicrobial resistance in invasive non-typhoid *Salmonella* from the Democratic Republic of the Congo: emergence

of decreased fluoroquinolone susceptibility and extended-spectrum beta lactamases. *PLoS Negl. Trop. Dis.* 7**,** e2103.

Ma, L., Alba, J., Chang, F.-Y., Ishiguro, M., Yamaguchi, K., Siu, L.K., et al. (2005). Novel SHV-derived extended-spectrum β-lactamase, SHV-57, that confers resistance to ceftazidime but not cefazolin. *Antimicrob. Agents Chemother.* 49**,** 600-605.

Machado, E., Coque, T.M., Canton, R., Novais, A., Sousa, J.C., Baquero, F., et al. (2007). High diversity of extended-spectrum beta-lactamases among clinical isolates of *Enterobacteriaceae* from Portugal. *J. Antimicrob. Chemother.* 60**,** 1370-1374.

Machado, E., Coque, T.M., Cantón, R., Sousa, J.C., and Peixe, L. (2008). Antibiotic resistance integrons and extended-spectrum β-lactamases among *Enterobacteriaceae* isolates recovered from chickens and swine in Portugal. *J. Antimicrob. Chemother.* 62**,** 296-302.

Mansour, W., Dahmen, S., Poirel, L., Charfi, K., Bettaieb, D., Boujaafar, N., et al. (2009). Emergence of SHV-2a extended-spectrum β-lactamases in clinical isolates of *Pseudomonas aeruginosa* in a university hospital in Tunisia. *Microb. Drug Res.* 15**,** 295-301.

Maravic, A., Skocibusic, M., Cvjetan, S., Samanic, I., Fredotovic, Z., and Puizina, J. (2015). Prevalence and diversity of extended-spectrum-beta-lactamase-producing Enterobacteriaceae from marine beach waters. *Mar. Pollut. Bull.* 90**,** 60-67.

Maravić, A., Skočibušić, M., Šamanić, I., Fredotović, Ž., Cvjetan, S., Jutronić, M., et al. (2013). *Aeromonas* spp. simultaneously harbouring *bla*CTX-M-15, *bla*SHV-12, *bla*PER-1 and *bla*FOX-2, in wild-growing Mediterranean mussel (*Mytilus galloprovincialis*) from Adriatic Sea, Croatia. *Int. J. Food Microbiol.* 166**,** 301-308.

Markovska, R., Schneider, I., Keuleyan, E., Sredkova, M., Ivanova, D., Markova, B., et al. (2008). Extended-spectrum β-lactamase–producing *Enterobacteriaceae* in Bulgarian hospitals. *Microb. Drug Res.* 14**,** 119-128.

Mazzariol, A., Roelofsen, E., Koncan, R., Voss, A., and Cornaglia, G. (2007). Detection of a new SHV-type Extended-spectrum β-Lactamase, SHV-31, in a *Klebsiella pneumoniae* strain causing a large nosocomial outbreak in The Netherlands. *Antimicrob. Agents Chemother.* 51**,** 1082-1084.

Mendonça, N., Ferreira, E., and Caniça, M. (2006). Occurrence of a novel SHV-type enzyme (SHV-55) among isolates of *Klebsiella pneumoniae* from Portuguese origin in a comparison study for extended-spectrum β-lactamase–producing evaluation. *Diagn. Microbiol. Infect. Dis.* 56**,** 415-420.

Mendonça, N., Ferreira, E., Louro, D., and Caniça, M. (2009). Molecular epidemiology and antimicrobial susceptibility of extended- and broad-spectrum β-lactamase-producing *Klebsiella pneumoniae* isolated in Portugal. *Int. J. Antimicrob. Agents* 34**,** 29-37.

Morfín-Otero, R., Mendoza-Olazarán, S., Silva-Sánchez, J., Rodríguez-Noriega, E., Laca-Díaz, J., Tinoco-Carrillo, P., et al. (2013). Characterization of *Enterobacteriaceae* isolates obtained from a tertiary care hospital in Mexico, which produces extended-spectrum β-lactamase. *Microb. Drug Res.* 19**,** 378-383.

Mulvey, M.R., Bryce, E., Boyd, D., Ofner-Agostini, M., Christianson, S., Simor, A.E., et al. (2004). Ambler class A extended-spectrum beta-lactamase-producing *Escherichia coli* and *Klebsiella* spp. in Canadian hospitals. *Antimicrob. Agents Chemother.* 48**,** 1204-1214.

Muratani, T., Kobayashi, T., and Matsumoto, T. (2006). Emergence and prevalence of β-lactamase-producing *Klebsiella pneumoniae* resistant to cephems in Japan. *Int. J. Antimicrob. Agents* 27**,** 491-499.

Naas, T., Namdari, F., Réglier-Poupet, H., Poyart, C., and Nordmann, P. (2007). Panresistant extended-spectrum β-lactamase SHV-5-producing *Acinetobacter baumannii* from New York City. *J. Antimicrob. Chemother.* 60**,** 1174-1176.

Newire, E.A., Ahmed, S.F., House, B., Valiente, E., and Pimentel, G. (2013). Detection of new SHV-12, SHV-5 and SHV-2a variants of extended spectrum beta-lactamase in *Klebsiella pneumoniae* in Egypt. *Ann. Clin. Microbiol. Antimicrob.* 12**,** 1-12.

Nicolas, M.H., Jarlier, V., Honore, N., Philippon, A., and Cole, S.T. (1989). Molecular characterization of the gene encoding SHV-3 beta-lactamase responsible for transferable cefotaxime resistance in clinical isolates of *Klebsiella pneumoniae*. *Antimicrob. Agents Chemother.* 33**,** 2096-2100.

Noda, T., Murakami, K., Etoh, Y., Okamoto, F., Yatsuyanagi, J., Sera, N., et al. (2015). Increase in resistance to extended-spectrum cephalosporins in *Salmonella* isolated from retail chicken products in Japan. *PLoS One* 10**,** e0116927.

Nüesch-Inderbinen, M.T., Kayser, F.H., and Hächler, H. (1997). Survey and molecular genetics of SHV beta-lactamases in *Enterobacteriaceae* in Switzerland: two novel enzymes, SHV-11 and SHV-12. *Antimicrob. Agents Chemother.* 41**,** 943-949.

Pallecchi, L., Bartoloni, A., Fiorelli, C., Mantella, A., Di Maggio, T., Gamboa, H., et al. (2007). Rapid dissemination and diversity of CTX-M extended-spectrum β-lactamase genes in commensal *Escherichia coli* isolates from healthy children from low-resource settings in Latin America. *Antimicrob. Agents Chemother.* 51**,** 2720-2725.

Péduzzi, J., Barthélémy, M., Tiwari, K., Mattioni, D., and Labia, R. (1989). Structural features related to hydrolytic activity against ceftazidime of plasmid-mediated SHV-type CAZ-5 beta-lactamase. *Antimicrob. Agents Chemother.* 33**,** 2160-2163.

Pinto, L., Radhouani, H., Coelho, C., Martins Da Costa, P., Simões, R., Brandão, R.M.L., et al. (2010). Genetic detection of extended-spectrum β-lactamase-containing *Escherichia coli* isolates from birds of prey from Serra da Estrela Natural Reserve in Portugal. *Appl. Environ. Microbiol.* 76**,** 4118-4120.

Pitout, J.D.D., Thomson, K.S., Hanson, N.D., Ehrhardt, A.F., Coudron, P., and Sanders, C.C. (1998). Plasmid-mediated resistance to expanded-spectrum cephalosporins among *Enterobacter aerogenes* strains. *Antimicrob. Agents Chemother.* 42**,** 596-600.

Podbielski, A., Schönling, J., Melzer, B., Warnatz, K., and Leusch, H.-G. (1991). Molecular characterization of a new plasmid-encoded SHV-type β-lactamase (SHV-2 variant) conferring high-level cefotaxime resistance upon *Klebsiella pneumoniae*. *J. Gen. Microbiol.* 137**,** 569-578.

Poirel, L., Héritier, C., Podglajen, I., Sougakoff, W., Gutmann, L., and Nordmann, P. (2003). Emergence in *Klebsiella pneumoniae* of a chromosome-encoded SHV β-Lactamase that compromises the efficacy of imipenem. *Antimicrob. Agents Chemother.* 47**,** 755-758.

Poirel, L., Lebessi, E., Castro, M., Fèvre, C., Foustoukou, M., and Nordmann, P. (2004). Nosocomial outbreak of extended-spectrum β-lactamase SHV-5-producing isolates of *Pseudomonas aeruginosa* in Athens, Greece. *Antimicrob. Agents Chemother.* 48**,** 2277-2279.

Politi, L., Tassios, P.T., Lambiri, M., Kansouzidou, A., Pasiotou, M., Vatopoulos, A.C., et al. (2005). Repeated occurrence of diverse extended-spectrum β-lactamases in minor serotypes of food-borne *Salmonella enterica* subsp. *enterica*. *J. Clin. Microbiol.* 43**,** 3453-3456.

Pouget, J.G., Coutinho, F.J., Reid-Smith, R.J., and Boerlin, P. (2013). Characterization of *bla*SHV genes on plasmids from *Escherichia coli* and *Salmonella enterica* isolates from Canadian food animals (2006-2007). *Appl. Environ. Microbiol.* 79**,** 3864-3866.

Prinarakis, E.E., Tzelepi, E., Gazouli, M., Mentis, A.F., and Tzouvelekis, L.S. (1996). Characterization of a novel SHV α-lactamase variant that resembles the SHV-5 enzyme. *FEMS Microbiol. Lett.* 139**,** 229-234.

Ramdani-Bouguessa, N., Manageiro, V., Jones-Dias, D., Ferreira, E., Tazir, M., and Caniça, M. (2011). Role of SHV β-lactamase variants in resistance of clinical *Klebsiella pneumoniae* strains to β-lactams in an Algerian hospital. *J. Med. Microbiol.* 60**,** 983-987.

Rasheed, J.K., Anderson, G.J., Yigit, H., Queenan, A.M., Doménech-Sánchez, A., Swenson, J.M., et al. (2000). Characterization of the extended-spectrum β-lactamase reference strain, *Klebsiella pneumoniae* K6 (ATCC 700603), which produces the Novel enzyme SHV-18. *Antimicrob. Agents Chemother.* 44**,** 2382-2388.

Rasheed, J.K., Jay, C., Metchock, B., Berkowitz, F., Weigel, L., Crellin, J., et al. (1997). Evolution of extended-spectrum beta-lactam resistance (SHV-8) in a strain of *Escherichia coli* during multiple episodes of bacteremia. *Antimicrob. Agents Chemother.* 41**,** 647-653.

Rocha-Gracia, R.C., Cortés-Cortés, G., Lozano-Zarain, P., Bello, F., Martínez-Laguna, Y., and Torres, C. (2015). Faecal *Escherichia coli* isolates from healthy dogs harbour CTX-M-15 and CMY-2 β-lactamases. *Vet. J.* 203**,** 315-319.

Sanchez-Romero, I., Asensio, A., Oteo, J., Munoz-Algarra, M., Isidoro, B., Vindel, A., et al. (2012). Nosocomial outbreak of VIM-1-producing *Klebsiella pneumoniae* isolates of multilocus sequence type 15: molecular basis, clinical risk factors, and outcome. *Antimicrob. Agents Chemother.* 56**,** 420-427.

Schooneveldt, J., Nimmo, G., and Giffard, P. (1998). Detection and characterisation of extended spectrum beta-lactamases in *Klebsiella pneumoniae* causing nosocomial infection. *Pathology* 30**,** 164-168.

Severin, J.A., Lestari, E.S., Kloezen, W., Lemmens-Den Toom, N., Mertaniasih, N.M., Kuntaman, K., et al. (2012). Faecal carriage of extended-spectrum β-lactamase-producing *Enterobacteriaceae* among humans in Java, Indonesia, in 2001–2002. *Trop. Med. Int. J.* 17**,** 455-461.

Shaheen, B.W., Nayak, R., Foley, S.L., Kweon, O., Deck, J., Park, M., et al. (2011). Molecular characterization of resistance to extended-spectrum cephalosporins in clinical *Escherichia coli* isolates from companion animals in the United States. *Antimicrob. Agents Chemother.* 55**,** 5666-5675.

Silva-Sanchez, J., Barrios, H., Reyna-Flores, F., Bello-Diaz, M., Sanchez-Perez, A., Rojas, T., et al. (2011). Prevalence and characterization of plasmid-mediated quinolone resistance genes in extended-spectrum β-lactamase–producing *Enterobacteriaceae* isolates in Mexico. *Microb. Drug Res.* 17**,** 497-505.

Song, W., Kim, J., Bae, I.K., Jeong, S.H., Seo, Y.H., Shin, J.H., et al. (2011). Chromosome-encoded AmpC and CTX-M extended-spectrum β-lactamases in clinical isolates of *Proteus mirabilis* from Korea. *Antimicrob. Agents Chemother.* 55**,** 1414-1419.

Song, W., Lee, K.M., Kim, H.-S., Kim, J.-S., Kim, J., Jeong, S.H., et al. (2006). Clonal spread of both oxyimino-cephalosporin- and cefoxitin-resistant *Klebsiella pneumoniae* isolates co-producing SHV-2a and DHA-1 β-lactamase at a burns intensive care unit. *Int. J. Antimicrob. Agents* 28**,** 520-524.

Stolle, I., Prenger-Berninghoff, E., Stamm, I., Scheufen, S., Hassdenteufel, E., Guenther, S., et al. (2013). Emergence of OXA-48 carbapenemase-producing *Escherichia coli* and *Klebsiella pneumoniae* in dogs. *J. Antimicrob. Chemother.* 68**,** 2802-2808.

Szabó, D., Bonomo, R.A., Silveira, F., Pasculle, A.W., Baxter, C., Linden, P.K., et al. (2005a). SHV-Type extended-spectrum beta-lactamase production is associated with reduced cefepime susceptibility in *Enterobacter cloacae*. *J. Clin. Microbiol.* 43**,** 5058-5064.

Szabó, D., Melan, M.A., Hujer, A.M., Bonomo, R.A., Hujer, K.M., Bethel, C.R., et al. (2005b). Molecular analysis of the simultaneous production of two SHV-type extended-spectrum beta-lactamases in a clinical isolate of *Enterobacter cloacae* by using single-nucleotide polymorphism genotyping. *Antimicrob. Agents Chemother.* 49**,** 4716-4720.

Tang, H.-J., Ku, Y.-H., Lee, M.-F., Chuang, Y.-C., and Yu, W.-L. (2015). In vitro activity of imipenem and colistin against a carbapenem-resistant *Klebsiella pneumoniae* isolate coproducing SHV-31, CMY-2, and DHA-1. *Biomed Res. Int.* 2015**,** 5.

Tasli, H., and Bahar, I.H. (2005). Molecular characterization of TEM- and SHV-derived extended-spectrum beta-lactamases in hospital-based *Enterobacteriaceae* in Turkey. *Jpn. J. Infect. Dis.* 58**,** 162-167.

Tian, G.-B., Wang, H.-N., Zhang, A.-Y., Zhang, Y., Fan, W.-Q., Xu, C.-W., et al. (2012). Detection of clinically important β-lactamases in commensal *Escherichia coli* of human and swine origin in western China. *J. Med. Microbiol.* 61**,** 233-238.

Timofte, D., Maciuca, I.E., Evans, N.J., Williams, H., Wattret, A., Fick, J.C., et al. (2014). Detection and molecular characterization of *Escherichia coli* CTX-M-15 and *Klebsiella pneumoniae* SHV-12 β-lactamases from bovine mastitis isolates in the United Kingdom. *Antimicrob. Agents Chemother.* 58**,** 789-794.

Tollentino, F.M., Polotto, M., Nogueira, M.L., Lincopan, N., Neves, P., Mamizuka, E.M., et al. (2010). High prevalence of *bla*CTX-M extended spectrum beta-lactamase genes in *Klebsiella pneumoniae* isolates

from a tertiary care hospital: first report of *bla*SHV-12, *bla*SHV-31, *bla*SHV-38, and *bla*CTX-M-15 in Brazil. *Microb. Drug Res.* 17**,** 7-16.

Uemura, S., Yokota, S.-I., Mizuno, H., Sakawaki, E., Sawamoto, K., Maekawa, K., et al. (2010). Acquisition of a transposon encoding extended-spectrum β-lactamase SHV-12 by *Pseudomonas aeruginosa* isolates during the clinical course of a burn patient. *Antimicrob. Agents Chemother.* 54**,** 3956-3959.

Usha, G., Chunderika, M., Prashini, M., Willem, S.A., and Yusuf, E.S. (2008). Characterization of extended-spectrum β-lactamases in *Salmonella* spp. at a tertiary hospital in Durban, South Africa. *Diagn. Microbiol. Infect. Dis.* 62**,** 86-91.

Vinue, L., Lantero, M., Saenz, Y., Somalo, S., De Diego, I., Perez, F., et al. (2008). Characterization of extended-spectrum beta-lactamases and integrons in *Escherichia coli* isolates in a Spanish hospital. *J. Med. Microbiol.* 57**,** 916-920.

Wang, M., Sahm, D.F., Jacoby, G.A., and Hooper, D.C. (2004). Emerging plasmid-mediated quinolone resistance associated with the *qnr* gene in *Klebsiella pneumoniae* clinical isolates in the United States. *Antimicrob. Agents Chemother.* 48**,** 1295-1299.

Whichard, J., Gay, K., Stevenson, J., Joyce, K., Cooper, K., Omondi, M., et al. (2007). Human *Salmonella* and concurrent decreased susceptibility to quinolones and extended-spectrum cephalosporins. *Emerg. Infect. Dis.* 13**,** 1681.

Yang, H.-F., Cheng, J., Hu, L.-F., Ye, Y., and Li, J.-B. (2012). Plasmid-mediated quinolone resistance in extended-spectrum-β-lactamase- and AmpC β-lactamase-producing *Serratia marcescens* in China. *Antimicrob. Agents Chemother.* 56**,** 4529-4531.

Yigit, H., Queenan, A.M., Rasheed, J.K., Biddle, J.W., Domenech-Sanchez, A., Alberti, S., et al. (2003). Carbapenem-resistant strain of *Klebsiella oxytoca* harboring carbapenem-hydrolyzing beta-lactamase KPC-2. *Antimicrob. Agents Chemother.* 47**,** 3881-3889.

Yu, Y., Ji, S., Chen, Y., Zhou, W., Wei, Z., Li, L., et al. (2007). Resistance of strains producing extended-spectrum β-lactamases and genotype distribution in China. *J. Infect.* 54**,** 53-57.

Yuan, M., Aucken, H., Hall, L.M., Pitt, T.L., and Livermore, D.M. (1998). Epidemiological typing of klebsiellae with extended-spectrum beta-lactamases from European intensive care units. *J. Antimicrob. Chemother.* 41**,** 527-539.

Yuan, M., Hall, L.M.C., Savelkoul, P.H.M., Vandenbroucke-Grauls, C.M.J.E., and Livermore, D.M. (2000). SHV-13, a novel extended-spectrum β-lactamase, in *Klebsiella pneumoniae* isolates from patients in an intensive care unit in Amsterdam. *Antimicrob. Agents Chemother.* 44**,** 1081-1084.

Zarnayová, M., Siebor, E., Péchinot, A., Duez, J.-M., Bujdáková, H., Labia, R., et al. (2005). Survey of *Enterobacteriaceae* producing extended-spectrum β-lactamases in a Slovak hospital: dominance of SHV-2a and characterization of TEM-132. *Antimicrob. Agents Chemother.* 49**,** 3066-3069.

Zou, L.-K., Wang, H.-N., Zeng, B., Zhang, A.-Y., Li, J.-N., Li, X.-T., et al. (2011). Phenotypic and genotypic characterization of β-lactam resistance in *Klebsiella pneumoniae* isolated from swine. *Vet. Microbiol.* 149**,** 139-146.

Zuo, B., Liu, Z., Wang, H., Yang, Y., Chen, J., and Ye, H. (2006). Genotype of TEM- and SHV-type beta-lactamase producing *Klebsiella pneumoniae* in Guangzhou area [Article in Chinese]. *Zhonghua Yi Xue Za Zhi* 86**,** 2928-2932.

Zurfluh, K., Hachler, H., Nuesch-Inderbinen, M., and Stephan, R. (2013). Characteristics of extended-spectrum beta-lactamase- and carbapenemase-producing Enterobacteriaceae Isolates from rivers and lakes in Switzerland. *Appl. Environ. Microbiol.* 79**,** 3021-3026.

Zurfluh, K., Nüesch-Inderbinen, M., Morach, M., Zihler Berner, A., Hächler, H., and Stephan, R. (2015). Extended-spectrum-β-lactamase-producing *Enterobacteriaceae* isolated from vegetables imported from the Dominican Republic, India, Thailand, and Vietnam. *Appl. Environ. Microbiol.* 81**,** 3115-3120.
