# Supplementary material for: A Review of SHV Extended-Spectrum β-Lactamases: Neglected Yet Ubiquitous
Source: Front Microbiol. 2016 Sep 5;7:1374. doi: 10.3389/fmicb.2016.01374 (PMC5011133; doi:10.3389/fmicb.2016.01374)
Supplement: Supplementary file 2 [file Image1.PDF]

Figure S1. Maximum likelihood nucleotide tree of 142 SHV-type β-lactamases. Variants whose sequence was not released in GenBank as of June 2016, that showed partial sequence or were identical to others (http://www.lahey.org/studies/) were not included in the analysis. $bla_{SHV-180}$ and $bla_{SHV-181}$ share the same sequence, as well as $bla_{SHV-121}$ and $bla_{SHV-136}$. The tree was implemented in Mega version 6.06 (Tamura *et al.*, 2013). Solid circles represent: red, extended-spectrum β-lactamases (2be; *n*=39); green, broad-spectrum β-lactamases (2br, *n*=5); and blue, penicillinases (2b, *n*=30). Unclassified alleles are reported in black (*n*=68).

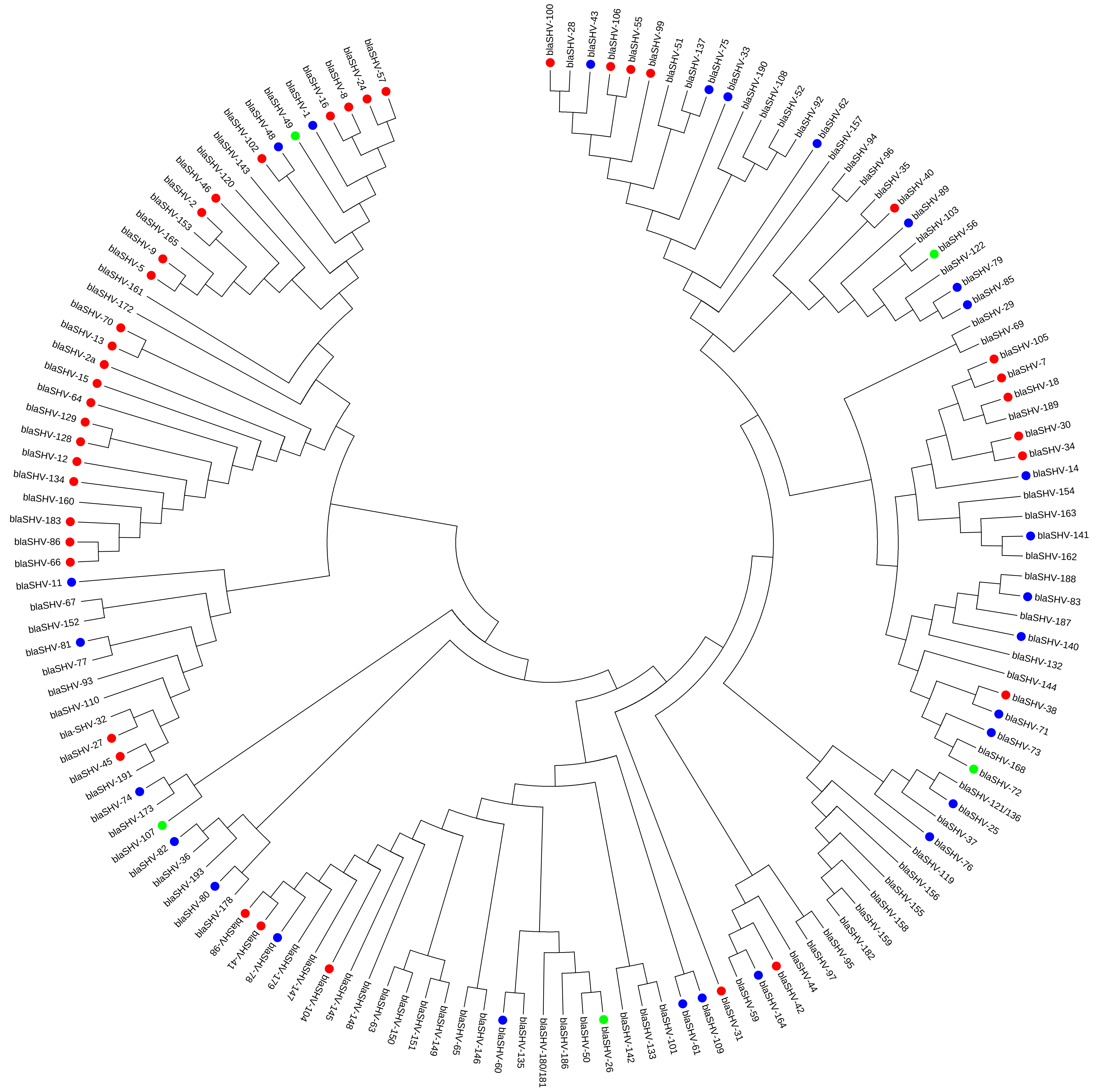

blaSHV-100
blaSHV-28
blaSHV-43
blaSHV-106
blaSHV-55
blaSHV-99
blaSHV-51
blaSHV-137
blaSHV-75
blaSHV-33
blaSHV-190
blaSHV-108
blaSHV-52
blaSHV-92
blaSHV-62
blaSHV-157
blaSHV-94
blaSHV-96
blaSHV-35
blaSHV-40
blaSHV-89
blaSHV-103
blaSHV-56
blaSHV-122
blaSHV-79
blaSHV-85
blaSHV-29
blaSHV-69
blaSHV-105
blaSHV-7
blaSHV-18
blaSHV-189
blaSHV-30
blaSHV-34
blaSHV-14
blaSHV-154
blaSHV-163
blaSHV-141
blaSHV-162
blaSHV-188
blaSHV-83
blaSHV-187
blaSHV-140
blaSHV-132
blaSHV-144
blaSHV-38
blaSHV-71
blaSHV-73
blaSHV-168
blaSHV-72
blaSHV-121/136
blaSHV-25
blaSHV-37
blaSHV-76
blaSHV-119
blaSHV-156
blaSHV-155
blaSHV-158
blaSHV-159
blaSHV-182
blaSHV-95
blaSHV-97
blaSHV-44
blaSHV-42
blaSHV-164
blaSHV-59
blaSHV-31
blaSHV-109
blaSHV-61
blaSHV-101
blaSHV-133
blaSHV-142
blaSHV-26
blaSHV-50
blaSHV-186
blaSHV-180/181
blaSHV-135
blaSHV-60
blaSHV-146
blaSHV-65
blaSHV-149
blaSHV-151
blaSHV-150
blaSHV-63
blaSHV-148
blaSHV-145
blaSHV-104
blaSHV-147
blaSHV-179
blaSHV-78
blaSHV-41
blaSHV-98
blaSHV-178
blaSHV-80
blaSHV-193
blaSHV-36
blaSHV-82
blaSHV-107
blaSHV-173
blaSHV-74
blaSHV-191
blaSHV-45
blaSHV-27
bla-SHV-32
blaSHV-110
blaSHV-93
blaSHV-77
blaSHV-81
blaSHV-152
blaSHV-67
blaSHV-11
blaSHV-66
blaSHV-86
blaSHV-183
blaSHV-160
blaSHV-134
blaSHV-12
blaSHV-128
blaSHV-129
blaSHV-64
blaSHV-15
blaSHV-2a
blaSHV-13
blaSHV-70
blaSHV-172
blaSHV-161
blaSHV-5
blaSHV-9
blaSHV-165
blaSHV-153
blaSHV-2
blaSHV-46
blaSHV-120
blaSHV-143
blaSHV-102
blaSHV-48
blaSHV-49
blaSHV-1
blaSHV-16
blaSHV-8
blaSHV-24
blaSHV-57
